# Cell adhesion molecule–related proteins signal to promote cytoneme elongation from Sonic Hedgehog–producing cells

**DOI:** 10.1126/sciadv.aei4501

**Published:** 2026-11-11

**Authors:** Christina A. Daly, Miriam E. Dillard, Eric T. Hall, Alfonso Lavado, Jonathon Klein, Jinbin Zhai, Shondra M. Pruett-Miller, Vishwajeeth R. Pagala, Anthony A. High, Alexandre F. Carisey, Christopher C. Hom, Nikki Koehler, Stacey K. Ogden

**Affiliations:** ^1^ Department of Cell and Molecular Biology, St. Jude Children’s Research Hospital, Memphis, TN 38105, USA.; ^2^ St. Jude Graduate School of Biomedical Sciences, St. Jude Children’s Research Hospital, Memphis, TN 38105, USA.; ^3^ Center for Pediatric Neurological Disease Research, St. Jude Children’s Research Hospital, Memphis, TN 38105, USA.; ^4^ Center for Advanced Genome Engineering, St. Jude Children’s Research Hospital, Memphis, TN 38105, USA.; ^5^ Center for Proteomics and Metabolomics, St. Jude Children’s Research Hospital, Memphis, TN 38105, USA.

## Abstract

Tissue morphogenesis and postdevelopmental homeostasis rely on efficient information exchange between communicating cells. Cytonemes are actin-based protrusions that contribute to precise information flow by facilitating contact-mediated directional transport of diverse signaling cargoes. How pathway-specific cytonemes are formed is poorly understood, as are the molecular mechanisms promoting actin organization for elongation and stability of the dynamic cellular extensions. We investigated biogenesis of Sonic Hedgehog (SHH) cytonemes, revealing functional repurposing of cell adhesion molecule-related proteins Brother of CDON (BOC) and Cell adhesion molecule-related, Down-regulated by Oncogenes (CDON) into recruiters of actin regulators FARP1 and Cdc42 for cytoneme elongation. Loss of BOC and CDON function blunts protrusion-localized Cdc42 activity, reducing cytoneme lengths and occurrence in SHH-expressing cells. Live imaging of developing murine spinal cords reveals that CDON loss in SHH-expressing cells reduces the frequency of neuroepithelial cytonemes and alters neuronal cell fate specification. These results inform a model whereby SHH-binding proteins promote ligand transport through stimulating formation of long cytonemes.

## INTRODUCTION

Embryonic tissue morphogenesis and postdevelopmental homeostasis depend on temporally and spatially controlled intercellular signals that are conveyed through morphogens and growth factors. Morphogens act in time- and concentration-dependent manners to govern cell fate, and growth factors signal at short and long distance to instruct cellular growth, division, and differentiation (*1*). Dysregulation of morphogen or growth factor–regulated pathways can trigger developmental disorders or cancers, underscoring the importance of tight control of signal deployment, reception, and interpretation during tissue development and maintenance (*2*–*6*).

One mechanism by which cells precisely control delivery of crucial information is by extending dedicated transport filopodia called cytonemes. These structures are actin-based, long, thin, and dynamic, and can originate from both signal-producing and -receiving cells (*7*, *8*). Cytonemes have been observed during tissue development and in the organization and maintenance of stem cell niches in *Drosophila*, zebrafish, chick, axolotl, and mammalian model systems (*9*–*15*). Cell-based assays suggest that human cells also form cytonemes, supporting broadly conserved use of the specialized signaling filopodia (*16*).

Components of Hedgehog (Hh)/Sonic Hedgehog (SHH), Wingless (Wg)/WNT, bone morphogenic protein (BMP)/Decapentaplegic (Dpp), Notch, and fibroblast growth factor (FGF) signaling pathways can be transported along cytonemes across a range of cellular contexts (*7*, *10*, *11*, *13*, *17*–*21*). As facilitators of long-range signaling in developing and expanding tissues, cytonemes often extend to great lengths to reach their intended cellular targets (*7*, *14*). In stem cell niches, cytoneme signaling promotes self-reinforcing regulatory activity and also provides positional information that dictates differentiation and de-differentiation for niche organization (*19*, *22*). This highlights their ability to adapt their function for context-dependent needs.

Cytoneme biogenesis and cargo specificity are tightly controlled such that individual extensions typically restrict themselves to transport of one type of signaling protein or receptor at a time (*10*, *23*–*25*). For example, during wing disc development in *Drosophila*, cells of the air sac primordium (ASP) extend spatially distinct cytonemes that either contain the FGF receptor Branchless (Bnl) or the Dpp receptor Thickveins (Tkv) (*23*). Similarly, wing disc-localized myoblasts form dedicated cytonemes that either transport the Notch ligand Delta to the ASP or receive Wg from the wing disc (*24*). In NIH3T3 cells, expression of SHH, WNT, FGF2, or the Notch ligand Jagged-1 correlates with increased cytoneme occurrence rates (*26*), suggesting that cargo-specific signals linked to either ligand production or autocrine activity might induce cytoneme formation. Consistent with this hypothesis, published reports indicate that cargo proteins must communicate with actin regulators for increased cytoneme biogenesis to occur (*11*, *27*–*29*). A knowledge gap remains in understanding of how such factors are recruited in pathway-specific manners to sites of cytoneme initiation.

Studies focused on WNT and SHH cytonemes have begun to close this knowledge gap by revealing that distinct transmembrane ligand-binding proteins can enhance cytoneme formation. WNT8a functions with its co-receptor receptor tyrosine kinase-like orphan receptor 2 (ROR2) and the planar cell polarity protein Vangl2 to promote cytoneme elongation in a Cdc42, N-WASP, and I-BAR protein (IRSp53)–dependent manner (*8*, *28*, *29*). Similarly, the SHH-binding, immunoglobulin super family molecules Cell adhesion molecule-related, Down-regulated by Oncogenes (CDON) and Brother of CDON (BOC) have been shown to localize to cytonemes and promote their formation (*26*, *30*). Overexpression of either protein drives ectopic cytoneme occurrence in the absence of SHH, and their combined removal prevents SHH-mediated cytoneme enhancement (*26*).

The *Drosophila* ortholog of CDON, interference hedgehog (Ihog), can stabilize Hh cytonemes when overexpressed (*17*, *31*). This activity is dependent on the Ihog fibronectin (FNIII) domains, which engage in trans homophilic interactions that link cytonemes along their lengths (*32*). Ihog has also been shown to bind glypicans in the extracellular milieu to guide and support cytonemes as they elongate (*32*, *33*). The intracellular domain of Ihog recruits the actin cross-linker Cheerio (filamin A), suggesting that the co-receptor can stabilize cytonemes through both extracellular and intracellular interactions (*34*). Whereas adhesion/homophilic activity by the BOC and CDON extracellular domains (ECDs) has not yet been demonstrated, the adhesion properties of Ihog suggest that they may be capable of cell adhesion molecule (CAM)–like activity in SHH cytonemes.

The ability of BOC and CDON to influence SHH cytoneme dynamics is notable because they were first proposed to function in SHH-receiving cells as co-receptors, directly binding to both SHH and its receptor Patched (PTCH) to facilitate pathway activation (*35*, *36*). Subsequent models indicated that BOC and CDON cooperate with a third, GPI-linked, co-receptor, growth arrest specific 1 (GAS1), whereby BOC and CDON cluster ligand on the cell surface and GAS1 transfers it to PTCH for pathway activation (*37*, *38*). As such, the discovery of their ability to promote cytoneme occurrence suggests context-specific diversification of their function in the SHH signaling pathway.

SHH deployment from ligand-producing cells requires Dispatched 1 (DISP), a 12-pass transmembrane protein that is structurally related to PTCH (*39*–*41*). DISP promotes transport of SHH through at least two mechanisms: (i) by transferring it to the secreted glycoprotein SCUBE2, which is reported to function as an extracellular SHH chaperone, and (ii) by enhancing SHH loading into cytonemes for protrusion-based transport (*10*, *42*, *43*). DISP can also bind BOC and CDON in SHH-producing cells where it colocalizes with them at distinct puncta along SHH-transporting cytonemes (*26*, *30*). This suggests that BOC and/or CDON associate with SHH-bound DISP in ligand-producing cells to form an SHH deployment complex (SDC).

Herein, we interrogate the function of the SDC in vitro and in vivo and map a signaling cascade that is activated by BOC or CDON to facilitate SHH cytoneme elongation. This BOC/CDON-initiated signal instructs cytoneme growth from the ligand source cells through recruitment of the guanine nucleotide exchange factor (GEF) FERM, RhoGEF, and Pleckstrin domain-containing protein 1 (FARP1) for Cdc42 activation. This activity is distinct from the well-established receiving cell signaling mechanism by which BOC and CDON promote activation of the signal transducing protein Smoothened (SMO) in SHH-stimulated cells through their function in the PTCH co-receptor complex (*35*, *36*, *38*). Live imaging of murine neural tubes reveals that in vivo loss of *Cdon* specifically in SHH-producing neuroepithelial cells reduces cytoneme length and extension frequency and results in altered specification of ventral neural progenitor cell populations. Altogether, the results presented here support a crucial role for SHH binding, CAM-related proteins as conduits for actin organization into cytonemes that ensure morphogen gradient precision.

## RESULTS

### SHH induces cytoneme formation through BOC and CDON

DISP and PTCH are 12-pass transmembrane proteins that bind SHH in signal-producing and -receiving cells, respectively. DISP is required for SHH release from ligand-producing cells while PTCH receives SHH ligand for pathway activation (*44*–*46*). We previously showed that *Disp1*^−/−^ mouse embryonic fibroblasts (MEFs) (*44*) and *Ptch1*^−/−^ MEFs (*47*) are still capable of forming SHH-induced cytonemes (*26*). However, because these receptors are structurally similar and both able to bind SHH, we sought to test whether DISP and PTCH are compensating for one another in the single knockout cell lines. We used CRISPR-Cas9 on *Disp1*^−/−^ MEFs (*44*) to generate *Disp1*/*Ptch1*/*Ptch2* triple knockout (TKO) MEFs, enabling direct comparison of the cell lines (fig. S1A). Knockout lines generated in this study were validated by testing for expression of the targeted gene and by functional assays, as appropriate (fig. S1, B to G). We evaluated the cytoneme-forming potential of *Disp1*/*Ptch1*/*Ptch2*^−/−^ MEFs following expression of SHH by quantifying the percentage of cells extending cytonemes in control and ligand-stimulated populations. We considered a cellular extension to be a cytoneme if it was thin (∼200 nm), was ≥10 μm in length, appeared to be closed ended, and, in assays with fluorescently tagged ligand, contained SHH (*48*). Consistent with our previous report (*26*), the single gene knockout cell lines remained responsive to SHH expression, increasing cytoneme occurrence similar to wild-type (WT) control MEFs. Similarly, *Disp1*/*Ptch1*/*Ptch2*^−/−^ TKO MEFs maintained the ability to increase cytoneme occurrence rates in response to SHH expression (Fig. 1A). These results confirm that neither PTCH nor DISP is required for cytoneme outgrowth from SHH-producing cells.

**Fig. 1.**
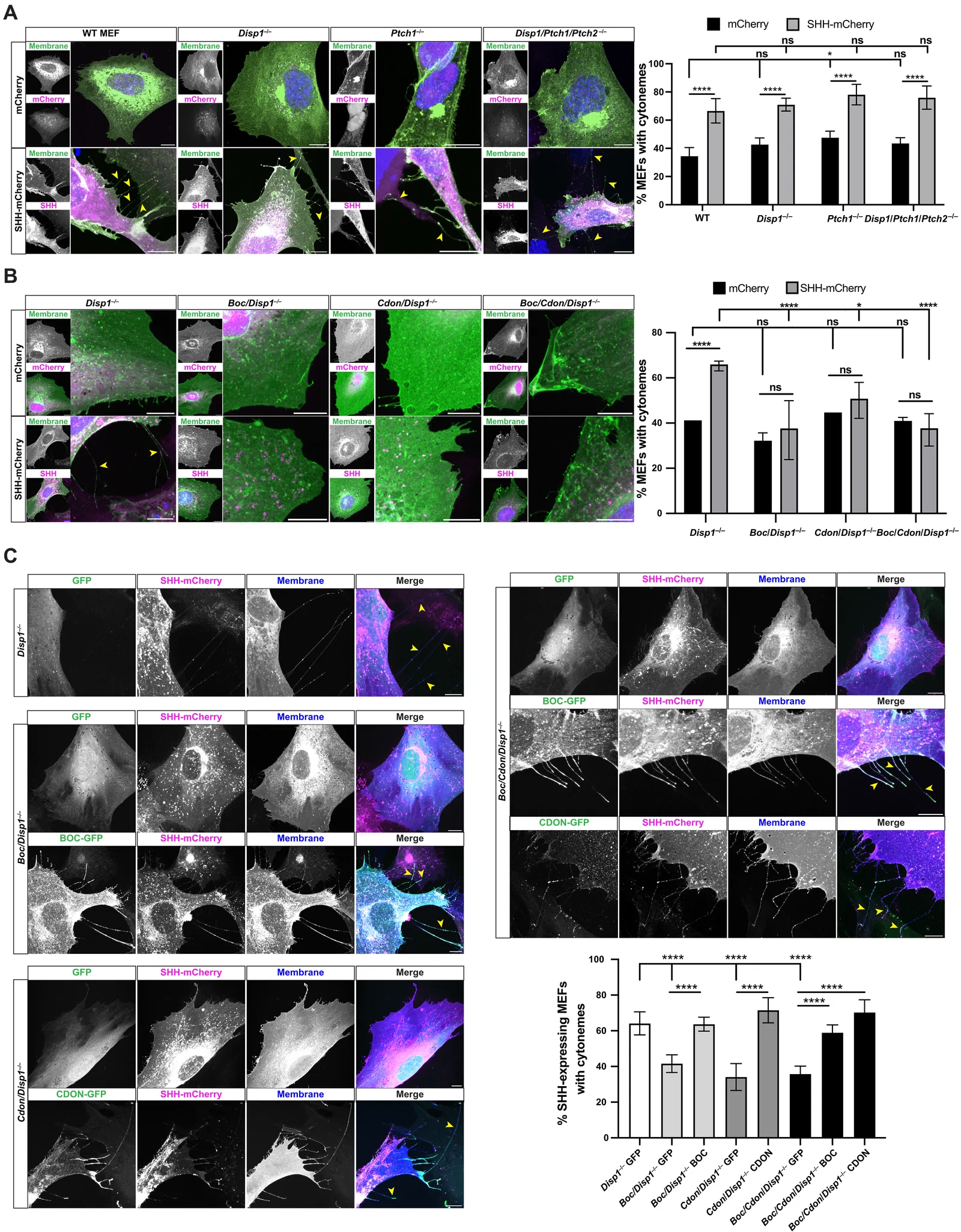
BOC and CDON facilitate SHH cytoneme induction. (**A**) Immunofluorescence of WT, *Disp1*^−/−^, *Ptch1*^−/−^, and *Disp1*/*Ptch1*/*Ptch2*^−/−^ MEFs expressing membrane pHluorin (green) and mCherry or full-length SHH (SHH)–mCherry (magenta) with DAPI shown in blue. Cytoneme occurrence rates were quantified on three biological replicates totaling 166 to 279 cells per condition. (**B**) Immunofluorescence of *Disp1*^−/−^, *Boc*/*Disp1*^−/−^, *Cdon*/*Disp1*^−/−^, and *Boc*/*Cdon*/*Disp1*^−/−^ MEFs expressing membrane-pHluorin (green) and mCherry or SHH-mCherry (magenta) with DAPI in blue. Percentage of cytoneme occurrence was quantified across four biological replicates (*n* = 242 to 368 cells per condition). (**C**) *Disp1*^−/−^, *Boc*/*Disp1*^−/−^, *Cdon*/*Disp1*^−/−^, and *Boc*/*Cdon*/*Disp1*^−/−^ MEFs were transfected with membrane-BFP (blue), SHH-mCherry (magenta), and GFP, BOC-GFP, or CDON-GFP (green). Percentage of cytoneme occurrence was quantified over three biological replicates totaling *n* = 248 to 358 cells per condition. Arrowheads mark cytonemes. Scale bars, 10 μm. Data shown as mean ± SD. Significance was calculated using one-way ANOVA. *P < 0.05, ****P < 0.0001; ns, not significant.

To evaluate the requirement of additional SDC components for initiation of SHH cytonemes, we used CRISPR-Cas9 to generate *Boc*/*Disp1*^−/−^ double knockout (DKO), *Cdon*/*Disp1*^−/−^ DKO, and *Boc*/*Cdon*/*Disp1*^−/−^ TKO MEFs (fig. S1, A and C) from *Disp1^−/−^* cells (*44*). Loss of BOC or CDON individually or in combination on the *Disp1*^−/−^ background reduced SHH cytoneme induction (Fig. 1B). To test whether expression of exogenous BOC or CDON could rescue cytoneme occurrence rates in the knockout cells, we expressed GFP control or GFP-tagged BOC or CDON proteins in SHH-expressing knockout cells and then quantified cytoneme occurrence. Individual overexpression of either BOC or CDON restored cytoneme outgrowth in SHH-expressing knockout lines (Fig. 1C, gray columns). Whereas combined loss of either *Boc* or *Cdon* with *Disp1* disrupted the ability of SHH to induce cytonemes in the DKO lines, individual add back of either BOC or CDON was sufficient to rescue cytoneme occurrence rates in *Boc/Cdon/Disp1^−/−^* TKO MEFs (Fig. 1B compared to Fig. 1C, black columns). This suggests a requirement for both CAM-related proteins to form a functional SDC for cytoneme outgrowth when they are expressed at endogenous levels in the absence of *Disp1*, but that when overexpressed, either BOC or CDON can compensate for loss of the other SDC components to drive cytoneme formation (Fig. 1C). Nonetheless, these results support that either BOC or CDON is required for the SDC to promote cytoneme biogenesis.

Given our observations that BOC and CDON promote cytoneme occurrence in SHH-expressing cells (Fig. 1) (*10*, *26*), we sought to specifically evaluate how their expression domains overlapped with cytoneme-producing cell populations in vivo. Thus, we performed RNAscope in situ hybridization (ISH) analysis on mouse embryonic neural tubes at E8.5 (10 somites), E9.5 (24 somites), and E10.5 (fig. S2A). Consistent with previous reports, *Boc* was broadly expressed in the neural tube, detectable in dorsal and intermediate cell populations at each time point evaluated (fig. S2A) (*37*, *49*). *Cdon* expression was more restricted, localizing to SHH-producing cell populations of the ventral neural tube including the notochord (E8.5 to E10.5) and floor plate (E9.5 to E10.5) (fig. S2A). Consistent with their documented contributions to neural crest migration in zebrafish (*49*, *50*), both *Boc* and *Cdon* were expressed in cells surrounding the dorsal roof plate at all time points evaluated, with *Cdon* expression being particularly intense near the roof plate at E10.5 (fig. S2A). Immunohistochemistry (IHC) of E9.5 neural tubes revealed that protein expression matched gene expression patterns for both BOC and CDON (fig. S2B).

The expression domains of *Boc* and *Cdon* were noteworthy to us for two reasons: (i) Floor plate and notochord-localized cells extend SHH-positive cytonemes that contact ventral cell populations, and (ii) cells of the roof plate, which produce WNT1/3a, extend actin-positive protrusions ventrally (*10*). We therefore hypothesized that, in addition to influencing SHH cytonemes, BOC and CDON might also contribute to formation of WNT-transporting cytonemes. To test whether WNT-stimulated cytoneme occurrence was dependent on BOC or CDON, we expressed WNT1 in WT MEFs or MEFs lacking all three known SHH co-receptors—*Boc*, *Cdon*, and *Gas1* (*26*, *51*)—and then scored cytoneme occurrence rates in control and ligand-expressing cells. Because GAS1 does not influence cytoneme formation (*26*), we reasoned that any changes in protrusion occurrence in SHH or WNT-expressing *Boc*/*Cdon*/*Gas1*^−/−^ TKO cells would result from loss of BOC and/or CDON. Whereas the ability of SHH to induce cytonemes was attenuated by BOC and CDON loss, WNT1 increased cytoneme occurrence rates similarly in both WT cells and *Boc*/*Cdon*/*Gas1*^−/−^ TKO MEFs (fig. S2C). This suggests that the requirement for BOC or CDON for cytoneme regulation may be specific to SHH-containing cytonemes.

### CDON is required for SHH cytoneme biogenesis in the murine neural tube

Given its restricted expression domain and apparent specificity to SHH cytonemes, we next sought to determine whether CDON contributes to cytoneme biogenesis or elongation from cells of the embryonic notochord or neural tube floor plate. To do so, we performed live imaging of neural tube sections from *Shh-Cre;membrane-Tomato/membrane-GFP* (*mTmG*) reporter mice, which GFP labels membranes of *Shh*-expressing cells of the developing embryo (*9*, *10*). E9.5 embryos were collected and subjected to gentle vibratome sectioning, and then ventral neural tube sections were immediately imaged using live confocal fluorescence microscopy (Fig. 2A). Over the course of 10 min, numerous thin, GFP-positive, dynamic cellular protrusions were observed extending from cells of the ventral floor plate and reaching toward neighboring non–GFP-labeled cells (Fig. 2B, arrowheads, and movies S1 and S2). To determine whether these extensions were dependent on BOC or CDON, we introduced *Boc* knockout and *Cdon* flox alleles into *Shh-Cre;mTmG* reporter mice (*52*–*54*). This allowed generation of animals with conditional deletion of *Cdon* in *Shh-Cre*–expressing cells, with or without *Boc* knockout.

**Fig. 2.**
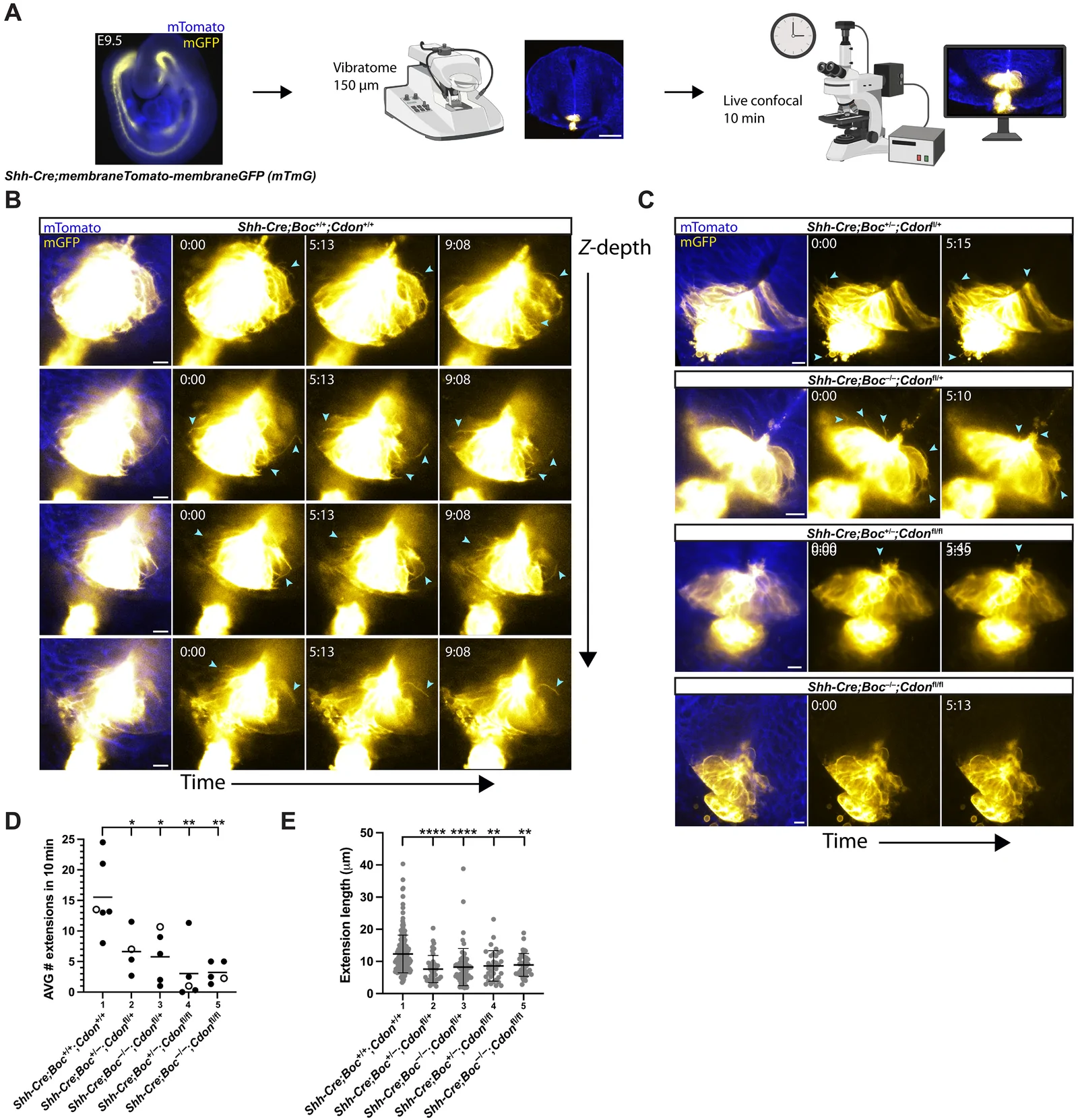
CDON stimulates cytoneme formation in the developing neural tube. (**A**) E9.5 *Shh-Cre*; *membrane-Tomato/membrane-GFP* (*mTmG*) embryos were vibratome sectioned at 150 μm thickness. Live imaging of neural tube floor plate and notochord in transverse sections was conducted for a minimum of 10 min. Membrane-Tomato is blue and membrane-GFP is yellow; scale bar, 100 μm. Created in BioRender. Daly, C. (2026) https://BioRender.com/ytmeufm. (**B**) Live imaging of a representative E9.5/26 somite stage *Shh-Cre;Boc^+/+^;Cdon^+/+^;mTmG* floor plate reveals numerous dynamic extensions (arrowheads) throughout the span of the section (*Z*-depth: top to bottom; timescale: left to right). Time stamp indicates minutes:seconds; scale bar, 10 μm. (**C**) Live imaging of representative E9.5/21 to 27 somite stage *Shh-Cre;mTmG, Boc*^+/−^ or *Boc*^−/−^, and *Cdon*^flox/+^ or *Cdon*^flox/flox^ neural tube sections shows that dynamic floor plate extensions (arrowheads) are reduced in the *Shh-Cre;Cdon^fl/fl^;mTmG* mutants (timescale: left to right; time stamp = minutes:seconds; scale bar, 10 μm). (**D**) Average number of floor plate extensions observed within a 10-min imaging period is calculated based on one to six sections per embryo, four to six embryos per genotype, with each embryo represented by a single point. Analysis was completed in a genotype-blinded manner. Embryos with representative images in (B) and (C) are plotted with open circles. (**E**) Individual extensions were measured at their maximum length achieved throughout the duration of imaging (34 to 183 extensions measured per genotype). *Shh-Cre;Boc^+/+^;Cdon^+/+^* embryos are 25 to 28 somites derived from three different litters. Mutant embryos are 20 to 28 somites and were littermates from five different litters. Data shown as mean (D) or mean ± SD (E). Significance was calculated using one-way ANOVA. *P < 0.05, ** *P* < 0.01, ****P < 0.0001; ns, not significant.

We confirmed specificity of the Cre deletion by evaluating neural tubes of control and experimental embryos using RNAscope and IHC analyses (fig. S2, D and E). Consistent with *Cre* activity being restricted to *Shh*-expressing cell populations, *Cdon* transcript was present in the dorsal neural tube and specifically reduced in the notochord and floor plate of *Shh-Cre;Cdon^fl/fl^* E9.5 embryos when compared to *Cre*-negative controls (fig. S2D, arrowhead). Despite some residual *Cdon* transcript in ventral-most cells of these neural tubes, likely due to *Cre* not being expressed until *Shh* promoter activity is induced in the floor plate around E9.0 (*55*), CDON protein was nearly undetectable in the ventral neural tube at E9.5. CDON protein levels remained high in the dorsal neural tube, further supporting specificity of *Cre* activity to *Shh*-expressing cells (fig. S2E).

Using this validated system, we analyzed cytoneme dynamics in *Shh-Cre;mTmG;Boc^+/−^;Cdon^fl/+^*, *Shh-Cre;mTmG;Boc^−/−^;Cdon^fl/+^*, *Shh-Cre;mTmG;Boc^+/−^;Cdon^fl/fl^*, and *Shh-Cre;mTmG;Boc^−/−^;Cdon^fl/fl^* embryos (Fig. 2C and movies S3 and S4). Cytoneme behavior was evaluated by scoring the average number of extensions observed over a 10-min window for each embryo (Fig. 2D) and by measuring the maximum length of each extension (Fig. 2E). Analyses were conducted in a blinded manner, with image acquisition and data quantification performed before receiving genotyping results. Consistent with the hypothesized role of CDON in ventral neural tube cytoneme formation, removal of one copy of *Cdon* reduced the average number and length of cytonemes independent of *Boc* status (Fig. 2, D and E, column 1 versus columns 2 and 3). Deletion of both copies of *Cdon* further reduced cytoneme numbers but did not lead to additional length reductions of extensions that did form (Fig. 2, D and E, columns 4 and 5).

SHH from the notochord and floor plate controls fate determination in ventral cell populations of the neural tube. The duration and concentration of SHH exposure dictate which transcriptional programs are activated, making specific transcription factors reliable markers for each cell population. These include, in a ventral to dorsal trajectory, FOXA2 in ventral-most cells of the floor plate, the high signal threshold target NKX2.2, intermediate ventral target OLIG2, PAX6 in the intermediate zone abutting the dorsal compartment, and PAX7 marking dorsal intermediate cell populations (*56*, *57*). Cross-repressive activities by these transcription factors ensure that each marker is restricted to the appropriate cell population (*58*). As such, analysis of the position and size of each marked cell population allows for direct reporting of SHH signaling gradient activity. To determine whether reduced cytoneme numbers and lengths resulting from *Cdon* deletion correlated with altered SHH gradient function, we sectioned E9.5 (∼25 somite) *Shh-Cre;Boc^+/−^*;Cdon*^fl/fl^* and *Shh-Cre;Boc^−/−^*;Cdon*^fl/fl^* embryos to quantify progenitor domain areas. *Boc^+/−^;Cdon^fl/fl^* lacking a *Cre* driver served as a control.

Notochord-derived SHH induces the FOXA2-positive floor plate, which functions as a secondary source of SHH protein for neural tube patterning (*59*). Suggestive of a role for notochord-derived CDON in deploying SHH to the floor plate, we observed a significant reduction in floor plate size relative to overall neural tube area in E9.5 *Shh-Cre;Boc^+/−^*;Cdon*^fl/fl^* and *Shh-Cre;Boc^−/−^;Cdon^fl/fl^* embryos when compared to the controls (Fig. 3, A and B, and fig. S2F). The NKX2.2 population, which requires high SHH pathway activity for its induction, showed a similarly reduced domain area compared to controls (Fig. 3, A, middle, and B, and fig. S2F). Whereas we did not observe the overall OLIG2 domain area to be significantly reduced compared to the controls, the neighboring PAX6 progenitor population was significantly expanded (Fig. 3, A and B, and fig. S2F). This is suggestive of reduced low-level SHH signaling activity in cells further from the ligand source (*58*, *60*). We attribute these changes to reduction in CDON activity in *Shh*-expressing cells, and not to loss of BOC in signal-receiving cells, because a similar PAX6 expansion is evident in *Shh-Cre;Boc^+/−^;Cdon^fl/fl^* embryos (Fig. 3B and fig. S2F) and *Boc^−/−^* neural tubes are phenotypically normal at E9.5 and E10.5 (*51*, *52*).

**Fig. 3.**
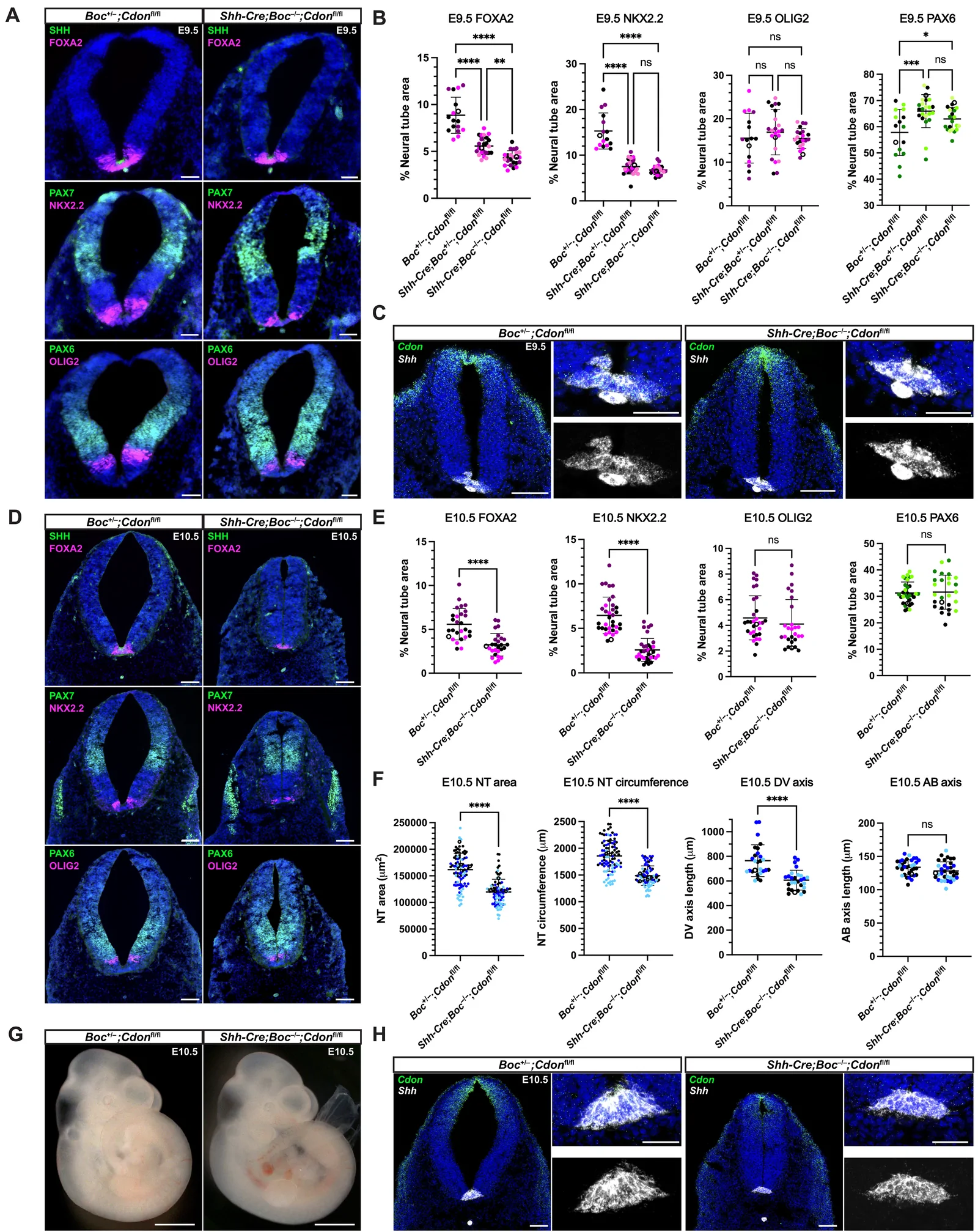
CDON loss in SHH-producing cells of the neural tube alters ventral fate specification. (**A**) E9.5/23 to 25 somite stage littermate embryos of indicated genotypes were sectioned and stained for the floor plate marker FOXA2 (magenta), SHH (green), and progenitor domain markers NKX2.2 (magenta), OLIG2 (magenta), PAX6 (green), and PAX7 (green); DAPI in blue. Cardiac-level sections are shown. Scale bars, 50 μm. (**B**) Progenitor domain size was quantified in respect to total neural tube area. Three to four somite-matched embryos from each genotype were examined. Sections from individual embryo pairs are indicated by different colored circles. Four to eight sections per embryo were analyzed. Open circles correspond to representative sections. (**C**) RNAscope in situ hybridization (ISH) of *Shh* (white) is shown in E9.5/25 somite stage embryos for the indicated genotypes. *Cdon* ISH is green and DAPI is blue. Scale bars, 100 μm for full neural tube images and 50 μm for floor plate zooms. (**D**) E10.5 cardiac-level sections of the indicated genotypes were stained for the same progenitor markers as in (A). Scale bars, 100 μm. (**E**) Progenitor domain area was quantified relative to total neural tube area for three somite-matched embryo pairs (color-matched). (**F**) Neural tubes were measured to quantify neuroepithelium area, neural tube circumference, and lengths of dorsoventral (DV, floor plate to roof plate) and apicobasal (AB, lumen to neural tube boundary) axes. (**G**) Whole embryo images corresponding to the sections in (D) and black circles in (E) and (F) demonstrate similar embryo morphology and size. Scale bars, 1 mm. (**H**) RNAscope ISH of *Shh* (white) and *Cdon* (green) at E10.5 in the indicated genotypes. DAPI is blue. Scale bars, 100 μm for full neural tube images and 50 μm for floor plate zooms. Data shown as mean ± SD. Significance was calculated using Student’s *t* test. *P < 0.05, **P < 0.01, ***P < 0.001, ****P < 0.0001; ns, not significant.

Relative size alterations for each of these neuroprogenitor populations remained consistent at E10.5 despite neural tubes being significantly smaller than control at this developmental stage (Fig. 3, D to F). Neural tube sizes were quantified for three somite-matched *Boc^+/−^;Cdon^fl/fl^* and *Shh-Cre;Boc^−/−^;Cdon^fl/fl^* embryo pairs, revealing reduced neural tube areas and circumferences in the *Cdon*-deleted mutants (Fig. 3F). Measurements of the dorsoventral (DV) and apicobasal (AB) axes demonstrated a marked reduction in DV growth, likely due to attenuated SHH signaling (Fig. 3F), which promotes proliferation and suppresses apoptosis along the DV axis (*61*, *62*). Nevertheless, the overall size of the embryos was comparable between control and *Cdon*-deleted animals, suggesting that the observed changes were likely specific to neural tube patterning defects and not a result of significantly delayed developmental timing in *Cre*-expressing embryos (Fig. 3G). Furthermore, the smaller mutant floor plates continued to express comparable levels of *Shh* transcript at E10.5 (Fig. 3H), supporting that the phenotypes observed were not due to impaired SHH production. Altogether, the observed alteration of high- and low-threshold SHH target gene expression suggests increased steepness of the signaling gradient following CDON protein reduction in notochord and floor plate cells. Combined with results presented above showing reduced numbers and lengths of neural tube cytonemes in *Cdon* conditionally deleted embryos, we conclude that disruption of the SHH signaling gradient likely results from reduced cytoneme-mediated SHH deployment from notochord and floor plate cells.

### BOC and CDON intracellular domains are required for cytoneme regulation

Having identified BOC and CDON as specific regulators of SHH cytoneme formation, we next sought to determine the molecular mechanisms by which they signal to influence cytoneme behavior. Both proteins contain extracellular Ig and FNIII domains that have the potential to facilitate intermolecular interactions in cis and trans (*63*, *64*). Accordingly, the *Drosophila* ortholog of CDON, Ihog, confers stability to cytonemes in wing imaginal discs through homophilic interactions (*32*, *33*). BOC and CDON also contain intracellular domains that facilitate communication with downstream actin regulatory effectors like Bnip-2, Cdc42, and members of MAPK and JNK pathways (*65*–*67*).

We chose to focus primarily on CDON because its expression in SHH-producing cells of the developing neural tube and the effects of its deletion in this population suggest a specific role in ligand delivery (*37*, *49*) (Figs. 2 and 3 and fig. S2). To test the requirement of CDON functional domains for cytoneme behavior, we generated deletion mutants lacking each domain. We engineered CDON proteins lacking the Ig domains (∆Ig), FNIII domains (∆FN), transmembrane domain (∆TM), or intracellular domain (∆ICD) and then tested for functional maturation and cell surface localization of each truncation mutant by analyzing N-linked glycosylation status and testing cell surface biotinylation (Fig. 4A and fig. S3, A to C). Cell surface biotinylation experiments showed that all truncation mutants, except for ∆TM, reached the cell surface (fig. S3A, lanes 6 to 8 and 10 versus lane 9). Accordingly, ∆Ig, ∆FN, and ∆ICD proteins all harbored complex N-linked glycans, as indicated by sensitivity to peptide-*N*-glycosidase F (PNGase), which cleaves both simple and complex N-glycan additions, and resistance to Endoglycosidase H (EndoH), which cleaves simple glycans that are present on ER-localized proteins (fig. S3, B and C). CDON∆Ig demonstrated modest mobility shifts in SDS-PAGE gels following EndoH versus PNGase treatments indicative of reduced glycosylation at baseline. This is likely due to deletion of multiple predicted N-linked glycosylation sites in the Ig domains (fig. S3, B and C, lanes 6 to 10). Consistent with its failure to reach the cell surface, ∆TM lacked EndoH-resistant complex glycans and immunofluorescence analysis of CDON∆TM-GFP-expressing *Boc/Cdon/Gas1^−/−^* MEFs showed subcellular localization suggestive of ER retention (fig. S3C, lanes 16 to 20, and Fig. 4B).

**Fig. 4.**
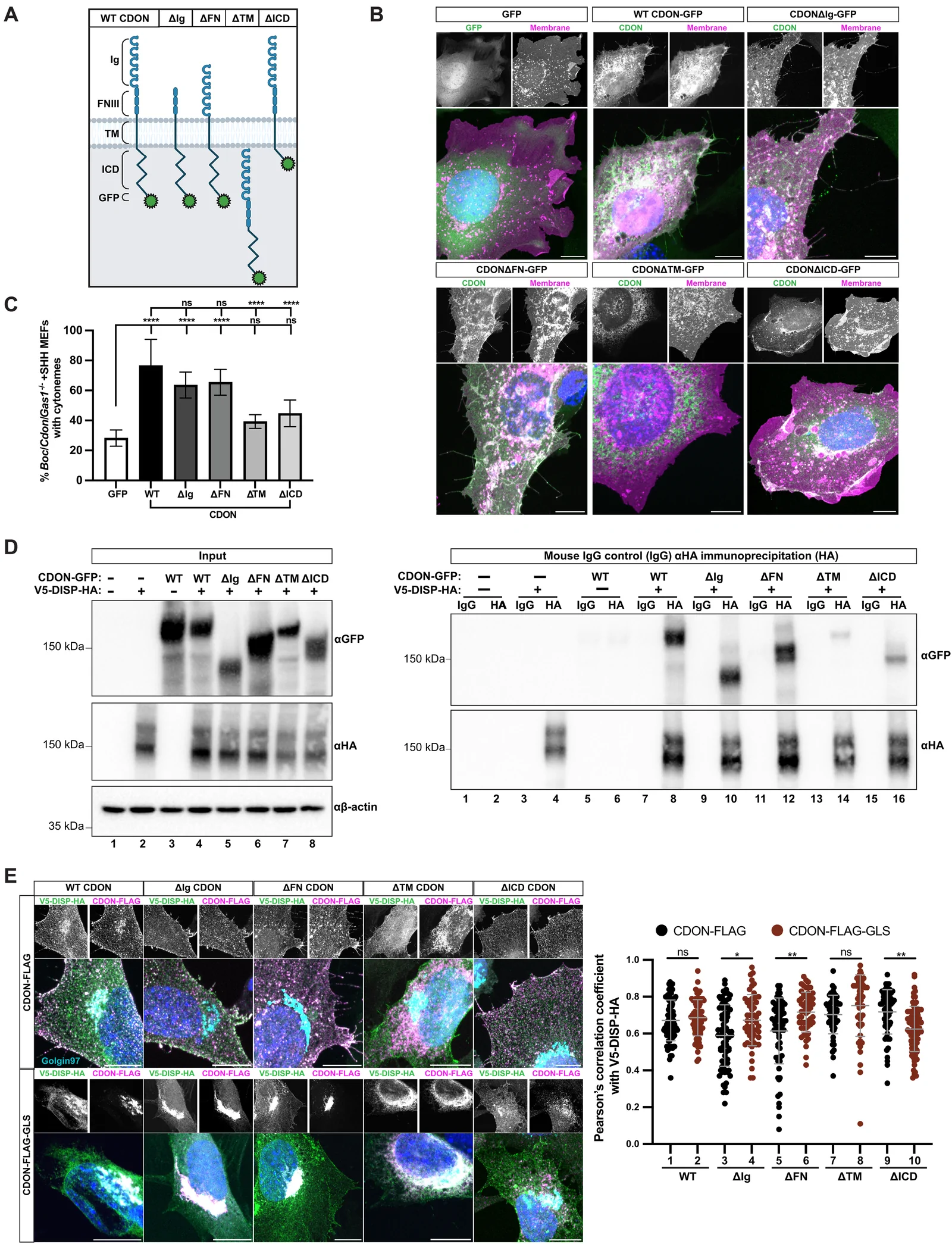
The CDON ICD is the preferred DISP binding domain. (**A**) CDON domain deletion mutants were generated to lack all five Ig domains (∆Ig), all three FNIII domains (∆FN), the transmembrane domain (∆TM), or the intracellular domain (∆ICD). Created in BioRender. Daly, C. (2026) https://BioRender.com/3cg2vkm. (**B**) *Boc*/*Cdon*/*Gas1*^−/−^ MEFs stably expressing SHH-FL were transfected with membrane-mCherry (magenta) and either GFP or the GFP-tagged CDON deletion mutants (green). DAPI is shown in blue. (**C**) Cytoneme occurrence rates were quantified over three biological replicates (*n* = 109 to 207 cells per condition). (**D**) Lysates prepared from HEK293T cells expressing V5-DISP-HA and/or GFP-tagged CDON deletion mutants were subjected to immunoprecipitation using αHA- or Mouse IgG-conjugated agarose. Input lysates and eluates were immunoblotted for GFP and HA. β-Actin serves as lysate loading control. Immunoblots are representative of two independent experiments. (**E**) Representative images of *Boc*/*Cdon*/*Gas1*^−/−^ MEFs transfected with V5-DISP-HA (HA in green) and either FLAG-tagged or Golgi localization sequence (GLS)–FLAG-tagged CDON deletion mutants (FLAG in magenta). DAPI is blue and Golgin-97 is cyan. Re-colocalization was quantified by Pearson’s colocalization coefficient within cell boundary region of interest (ROI) between HA and FLAG channels. Quantification was performed on images totaling *n* = 50 to 90 cells per condition taken over three biological replicates. Scale bars, 10 μm. Data shown as mean ± SD. Significance was calculated using one-way ANOVA. *P < 0.05, **P < 0.01, ****P < 0.0001; ns, not significant.

To survey the effects of CDON functional domain deletion on cytoneme dynamics, we expressed each protein in *Boc*/*Cdon*/*Gas1*^−/−^ TKO MEFs stably expressing MSCV-Hygro-SHH-FL (*26*, *51*) and then quantified cytoneme occurrence rates (Fig. 4, B and C). Whereas ∆Ig and ∆FN CDON proteins rescued cytoneme occurrence similarly to WT CDON, ∆TM and ∆ICD did not. The inability of CDON∆TM to rescue occurrence likely stems from its inability to effectively traffic out of the ER. As such, the cytoneme regulatory signal is likely communicated to downstream effectors through the CDON ICD.

To test whether the ICD is sufficient to promote cytoneme occurrence when localized to the plasma membrane, we generated a construct lacking the ECD, CDON∆ECD-GFP, and a chimeric protein where the CDON ECD was replaced by the ECD of CD4, a transmembrane protein with four tandem Ig domains (CD4::CDON) (fig. S4A). In an effort to achieve subcellular localization similar to WT CDON, we designed the two ECD mutant constructs to retain both the signal sequence and the FN-TM linker of WT CDON. Cell surface biotinylation experiments indicated that both ECD mutants reached the cell surface; however, CDON∆ECD was present on the surface at reduced levels as indicated by lower biotinylation signal (fig. S4B, lane 6 compared to lanes 4 and 5). PNGase and EndoH sensitivity assays revealed that WT CDON and CD4::CDON exhibited complex N-linked glycosylation (fig. S4C). CDON∆ECD was unaltered by EndoH or PNGase treatment (fig. S4C), consistent with removal of all predicted N-linked glycosylation sites in the Ig and FNIII domains (figs. S4C and S3B).

Having confirmed expression and plasma membrane localization of the CDON derivatives, we next assessed whether the mutant proteins localized to cytonemes or influenced their occurrence in *Boc/Cdon/Gas1^−/−^* MEFs. We found that CD4::CDON entered extensions like WT CDON and increased their occurrence over baseline, albeit to a lesser intensity than the WT protein (fig. S4D). This partial rescue was similar to what was observed with CDON∆Ig and CDON∆FN and suggests that most cytoneme-promoting activity can be conferred through the ICD if it can enter the specialized filopodia (Fig. 4C and fig. S4D). Accordingly, CDON∆ECD failed to localize as efficiently to cytonemes and showed a modest increase in cytoneme occurrence over baseline (fig. S4D). Altogether, these results suggest that enrichment in cytonemes is necessary for the ICD to signal for their elongation or occurrence. The intermediate cytoneme phenotype of CD4::CDON-GFP suggests that the CDON ECD contributes to promoting cytoneme occurrence.

Like CDON, BOC re-expression can rescue SHH-induced cytoneme stimulation in *Boc*/*Cdon*/*Gas1*^−/−^ MEFs (*26*). Thus, we tested whether BOC also signals to influence cytoneme behavior through its ICD. We generated BOC∆ICD-GFP, confirmed its cell surface localization and N-linked glycosylation status, and then tested its ability to promote cytoneme occurrence (fig. S5, A to E). Like what was observed for CDON, WT BOC was able to rescue SHH-stimulated cytoneme occurrence in *Boc*/*Cdon*/*Gas1*^−/−^ MEFs, but BOC∆ICD was not (fig. S5, D and E). Together with the results presented above, this suggests that the ICDs of BOC and CDON transduce a cytoneme-promoting signal.

### The CDON ICD facilitates DISP interaction

Structure/function analyses indicate that the SHH receptor PTCH associates with BOC/CDON through binding of its first large extracellular loop to BOC/CDON extracellular FNIII domains (*35*, *36*). Given that PTCH and the SDC member DISP are structurally related (*40*, *41*), we hypothesized that CDON binding to DISP in the SDC would occur through its ECDs. To test this, we coexpressed DISP with the CDON deletion mutants described above in human embryonic kidney (HEK) 293T cells and then performed coimmunoprecipitation analysis (Fig. 4D). Consistent with our previous report (*26*), GFP-tagged, full-length CDON specifically coimmunoprecipitated with V5-DISP-HA (Fig. 4D, IP lane 8 compared to lane 6). ∆Ig, ∆FN, ∆TM, and ∆ICD CDON-GFP deletion mutants were also found to coimmunoprecipitate with V5-DISP-HA, suggesting the presence of multiple binding interfaces (Fig. 4D, IP lanes 10, 12, 14, and 16). However, ∆TM and ∆ICD CDON proteins coimmunoprecipitated with V5-DISP-HA with reduced efficiency (Fig. 4D, lanes 14 and 16, top), suggesting that the ICD is likely a preferred interaction domain. We speculate that modest amount of ΔTM coimmunoprecipitating with V5-DISP-HA was likely due to failure of this truncation mutant to reach the cell surface (Fig. 4B and fig. S3A, lane 9).

To evaluate CDON/DISP interaction in cells, we performed Golgi re-colocalization assays (GRAs) (*68*). A C-terminal Golgi localization sequence (GLS) was fused to WT and each of the CDON-FLAG deletion mutants to tether them to the Golgi membrane (fig. S5F). V5-DISP-HA was expressed alongside these mutants in *Boc*/*Cdon*/*Gas1*^−/−^ MEFs and re-colocalization of WT DISP to the Golgi was monitored as an in-cell reporter of CDON-DISP association (Fig. 4E). Whereas robust Golgi localization of DISP was observed with WT CDON, ΔIg, and ΔFN CDON-GLS proteins, CDONΔICD-GLS failed to concentrate DISP at the Golgi (Fig. 4E, columns 1 to 8 versus columns 9 and 10). CDONΔICD-GLS was the only CDON variant to show reduced Pearson’s correlation coefficients with V5-DISP-HA when compared to the non-GLS control (Fig. 4E, column 9 versus column 10). We did not observe a significant change in colocalization between CDON∆TM and DISP, likely due to the inability of this mutant to effectively traffic from the ER (Fig. 4E, columns 7 and 8). Altogether, these results indicate that the ICD drives stable DISP-CDON association in cells.

### CDON and BOC recruit FARP1 to enable cytoneme occurrence

Because DISP cannot signal to influence cytoneme dynamics without BOC or CDON (*26*) (Fig. 1), we hypothesized that their ICDs bind actin regulatory effectors in addition to binding DISP. To test this in an unbiased manner, we generated BOC and CDON fusion proteins harboring a C-terminal intracellular FLAG tag followed by the BirA/TurboID biotin ligase (*69*) (fig. S6A). Unfortunately, the chimeric CDON-TurboID protein expressed poorly in cells, leading to insufficient biotinylation activity (fig. S6B, lane 4, control versus lanes 7 and 8, SAV). Thus, we conducted proximity labeling using BOC-TurboID. BOC-TurboID trafficked to the plasma membrane and harbored complex N-linked glycans comparable to the FLAG-tagged protein (fig. S6, C and D). To ensure that TurboID would not hinder functional interactions required for the cytoneme-promoting activity of BOC, we tested the ability of TurboID-containing BOC protein to rescue SHH cytoneme occurrence in *Boc*/*Cdon*/*Gas1*^−/−^ MEFs. The TurboID-tagged construct rescued SHH cytoneme occurrence comparable to the FLAG-tagged construct lacking the biotin ligase (fig. S6E). The chimeric protein was able to effectively biotinylate cellular proteins, as indicated by staining with fluorescently conjugated streptavidin (fig. S6E, SAV/cyan). Altogether, these assays support feasibility of using the chimeric BOC-FLAG-TurboID protein for proteomic experimentation (fig. S6, C to E).

In addition to its role in the SDC, BOC is important for clustering SHH on receiving cells for transfer to PTCH1 (*35*, *36*, *70*). BOC is also suggested to bind PTCH2 in some contexts, and PTCH2 can compensate for PTCH1 loss in the chick neural tube, raising the possibility that the presence of PTCH1/2 receptors could bias BOC away from SDC effectors (*71*–*73*). Thus, we generated *Boc*/*Cdon*/*Disp1*/*Ptch1*/*Ptch2*/*Smo*^−/−^ [pan receptor knockout (PRK)] MEFs to serve as a “clean” background for binding studies (fig. S1A). PRK cells were validated through loss of targeted gene transcripts, loss of induction of the SHH target *Gli1*, and inability to detect ligand-stimulated enrichment of the SHH signal transducing protein SMO in the primary cilium, a sensory organelle from which it signals (fig. S1, D to G). PRK cells were then transfected with TurboID or BOC-TurboID with or without SDC partners V5-DISP-HA and SHH. The cells were exposed to vehicle or biotin, and then biotinylated proteins were isolated from lysates on streptavidin-conjugated beads. Bound proteins were identified through LC-MS/MS using spectral counting-based quantification (fig. S6, F and G). We obtained high spectral counts for the coexpressed SDC components DISP and SHH, supporting that BOC-TurboID biotinylates known interaction partners (fig. S6G).

Given our hypothesis that the SDC communicates with cytoskeletal regulators to promote cytoneme initiation and/or elongation, we searched the dataset for direct actin regulators. We observed enrichment of members of the WAVE regulatory complex (fig. S6G), which is consistent with a recent report on BOC’s interaction with this complex in Rac1-driven actin polymerization for SHH-mediated axon guidance (*74*). To find potential cytoneme-promoting signaling molecules, we looked for Rho GTPases, which specifically alter actin dynamics at the plasma membrane (*75*). Although BOC proximity labeling did not enrich specific Rho GTPases, it did modestly enrich Rho activating GEFs (fig. S6G). We selected dedicator of cytokinesis 7 (DOCK7) and FARP1 for further analysis because of their established connections to actin regulation (*76*–*78*). We also included intersectin-1 (ITSN1), despite it showing low spectral counts in the BOC-TurboID dataset, because the intracellular domains of both BOC and CDON have polyproline motifs that can function as putative ITSN1 binding sites per Scansite 4.0 (*79*) (fig. S6H).

To test whether any of the candidate BOC interactors localize to cytonemes, we expressed DOCK7-GFP, FARP1-GFP, and ITSN1-mCherry in NIH3T3 cells and examined their subcellular localization. FARP1 was the only GEF that was detected in cytonemes, and it appeared to concentrate at cytoneme tips in SHH-expressing cells (Fig. 5A). Accordingly, FARP1-GFP was the only GEF that, when overexpressed in the absence of SHH, elevated baseline cytoneme occurrence rates (Fig. 5B). Whereas DOCK7-GFP had no impact on cytoneme occurrence rates, ITSN1-mCherry blocked the ability of SHH to increase cytoneme dynamics as compared to control (Fig. 5B). We speculate that ITSN1 overexpression may reduce SDC cell surface occupancy to prevent cytoneme biogenesis due to its ability to enable clathrin-mediated endocytosis (*80*–*82*). This is because DISP/SHH internalization occurs through clathrin-mediated endocytosis and both BOC and PTCH are internalized in an SHH-dependent manner through the clathrin adaptor Numb (*10*, *83*, *84*). As such, ITSN1, which directly interacts with Numb in neurogenesis (*85*), might serve as a general facilitator of SHH receptor endocytosis following ligand binding.

**Fig. 5.**
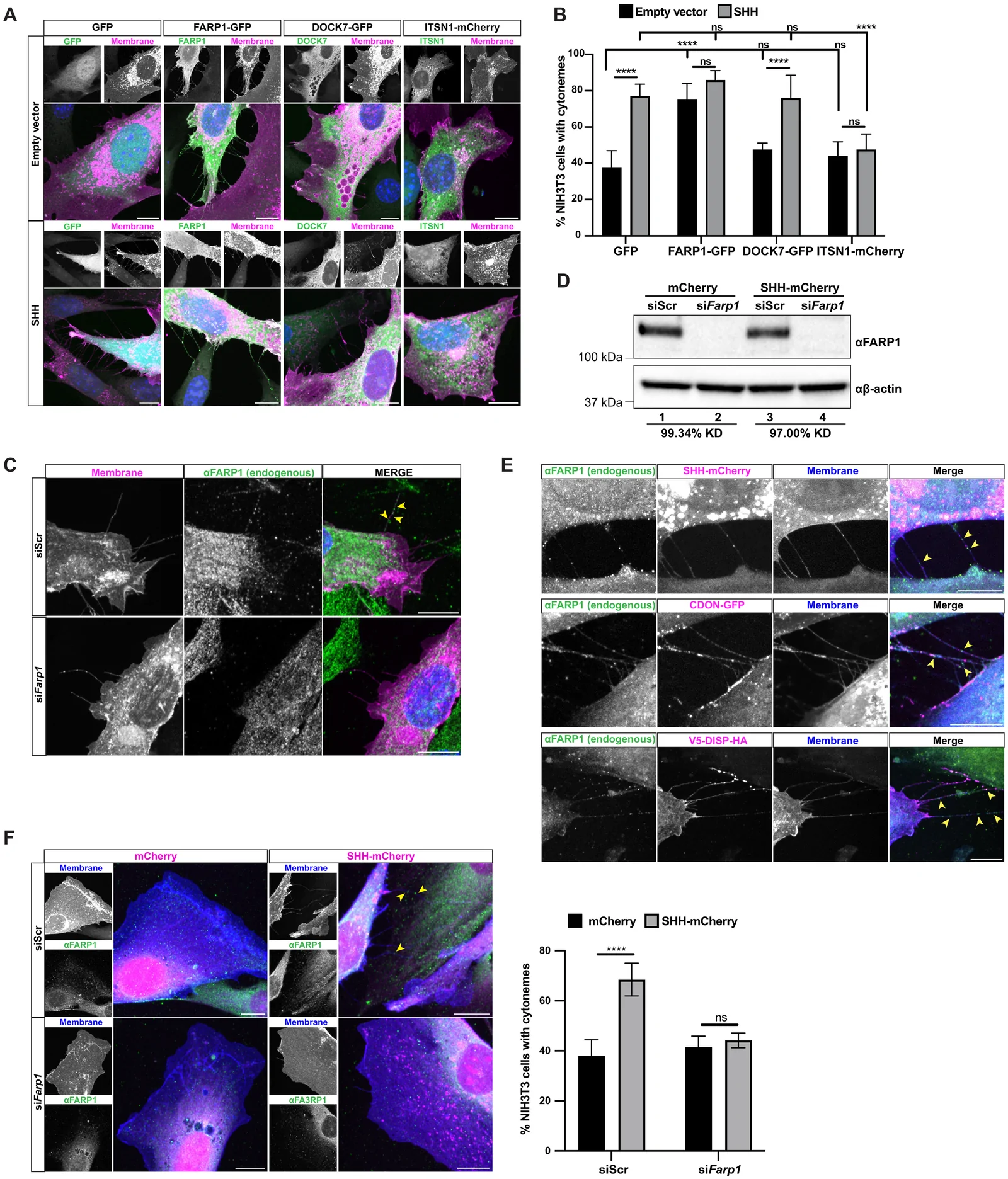
FARP1 promotes SHH cytoneme formation. (**A**) NIH3T3 cells stably expressing either MSCV-Hygro (empty vector) or MSCV-Hygro-SHH-FL (SHH) were transfected with either membrane-mCherry or membrane-pHluorin (magenta) and GFP, FARP1-GFP, DOCK7-GFP, or ITSN1-mCherry (green). DAPI is in blue. (**B**) Cytoneme occurrence rates were quantified across three biological replicates (*n* = 137 to 241 cells per condition). (**C** and **D**) *Farp1* knockdown in NIH3T3 cells expressing mCherry or SHH-mCherry and membrane-pHluorin [magenta in (C)] was validated by immunofluorescence (C) and immunoblotting (D) ∼48 hours after transfection with *Farp1* siRNA or scrambled control (siScr). β-Actin serves as a loading control for densitometry quantification of percent knockdown. Fixed cells were immunostained for endogenous FARP1 (green) with DAPI in blue. FARP1-positive puncta are evident in cell extensions, but absent when *Farp1* is knocked down (C, arrowheads). (**E**) NIH3T3 cells stably expressing MSCV-Hygro-SHH-FL were transfected with membrane-BFP (blue) and SHH-mCherry, CDON-GFP, or V5-DISP-HA (magenta), and then immunostained for endogenous FARP1 (green) following fixation. Endogenous FARP1-positive puncta are evident in cytonemes containing these SHH pathway members (arrowheads). (**F**) NIH3T3 cells were treated with si*Farp1* or siScr for 24 hours and then transfected with membrane-pHluorin (blue) and either mCherry or SHH-mCherry (magenta). Approximately 24 hours posttransfection, cells were fixed and stained for endogenous FARP1 (green). Cytoneme occurrence rates were quantified from three biological replicates totaling *n* = 248 to 316 cells per condition. Scale bars, 10 μm. Data shown as mean ± SD. Significance was calculated using one-way ANOVA. ****P < 0.0001; ns, not significant.

Given our observation that CDON promotes cytoneme occurrence in SHH-producing cells of the developing neural tube, we were keen to test whether CDON might interact with FARP1 in cells. Thus, we performed a proximity ligation assay (PLA) in NIH3T3 cells expressing CDON-FLAG and FARP1-GFP. We observed PLA signal between the two proteins along cytoneme lengths in both the absence and presence of SHH, and noted increased intensity of the PLA signal near cytoneme tips when SHH was coexpressed (fig. S7A), As a negative control, we tested for PLA activity between CDON-FLAG and a GFP-tagged, truncated GLI2 transcription factor construct that accumulates in the cytoplasm. GLI2-CDON-FLAG PLA signal was modest and rarely found within the CDON/SHH-induced extensions, supporting specificity of the CDON-FARP1 PLA interaction signal in cytonemes (fig. S7A).

To evaluate subcellular localization of endogenous FARP1, we immunostained NIH3T3 cells using anti-FARP1 and observed bright FARP1 puncta localized to cytoneme-like projections (Fig. 5C, yellow arrowheads). To test specificity of this signal, cells were transfected with scrambled or *Farp1* siRNA before immunostaining (Fig. 5, C and D). Whereas low-level background cytoplasmic staining was unaffected by *Farp1* knockdown, the bright puncta present on cellular extensions were lost, supporting specificity of the FARP1 cytoneme signal (Fig. 5C).

To determine whether endogenous FARP1 enriched with SHH, CDON, or DISP in cytonemes, SHH-mCherry, CDON-GFP, or V5-DISP-HA was expressed in NIH3T3 cells, and each protein was visualized along with FARP1. Endogenous FARP1-positive puncta were observed in SHH-mCherry, CDON-GFP, or V5-DISP-HA-positive cytonemes (Fig. 5E, yellow arrowheads), suggesting a functional contribution to cytoneme-based SDC activity. To test whether FARP1 is required for SHH cytoneme formation, we knocked down *Farp1* in NIH3T3 cells expressing SHH-mCherry and quantified cytoneme occurrence rates. SHH could not increase cytoneme occurrence over baseline following FARP1 knockdown, suggesting that the GEF is an essential downstream signaling effector of SDC regulation of cytoneme biogenesis or elongation (Fig. 5F).

### Cdc42 activation downstream of the SDC increases SHH cytoneme occurrence

FARP1 is a member of the Dbl GEF family that has been linked to dendritic spine formation and synaptogenesis in neurons (*77*, *78*, *86*, *87*). As a Dbl GEF, FARP1 structure includes tandem Dbl-Homology (DH) and pleckstrin homology (PH) domains, crucial for membrane targeting and substrate binding (*88*) (fig. S7B). FARP1 activity toward the Rho GTPases RhoA, Rac1, and Cdc42 is context dependent (*77*, *78*, *89*–*91*), necessitating empirical evaluation of which GTPase functions downstream of the GEF to influence cytoneme dynamics. Thus, we obtained constitutively active (CA), GFP-tagged RhoA, Rac1, and Cdc42 constructs, and then expressed each GTPase in NIH3T3 cells alone or with SHH-mCherry to evaluate the impact on cytoneme occurrence (Fig. 6, A and B). Only CA Cdc42 boosted baseline cytoneme occurrence rates. CA RhoA was not significantly different from the control cells, and, like ITSN1, CA Rac1 blunted the ability of SHH to induce cytonemes (Fig. 6B). CA Rac1 expression stimulated lamellipodia formation (Fig. 6A), suggestive of activation of effectors that oppose filopodial extension and instead promote assembly of branched actin ruffles through WAVE complex activity (*92*, *93*). These results suggest that Cdc42 may be activated downstream of BOC/CDON-FARP1 signaling to promote cytonemes. To test whether Cdc42 is required for SHH-stimulated cytoneme biogenesis, we transfected NIH3T3 cells with either mCherry or SHH-mCherry and then treated cells with either dimethyl sulfoxide (DMSO) or the specific Cdc42 inhibitor ML141 (*94*). Cytoneme occurrence rates were scored 1 hour after drug treatment. Suggestive of Cdc42 signaling downstream of SHH for cytoneme initiation, ML141 treatment eliminated the SHH effect on cytoneme occurrence rates (Fig. 6C).

**Fig. 6.**
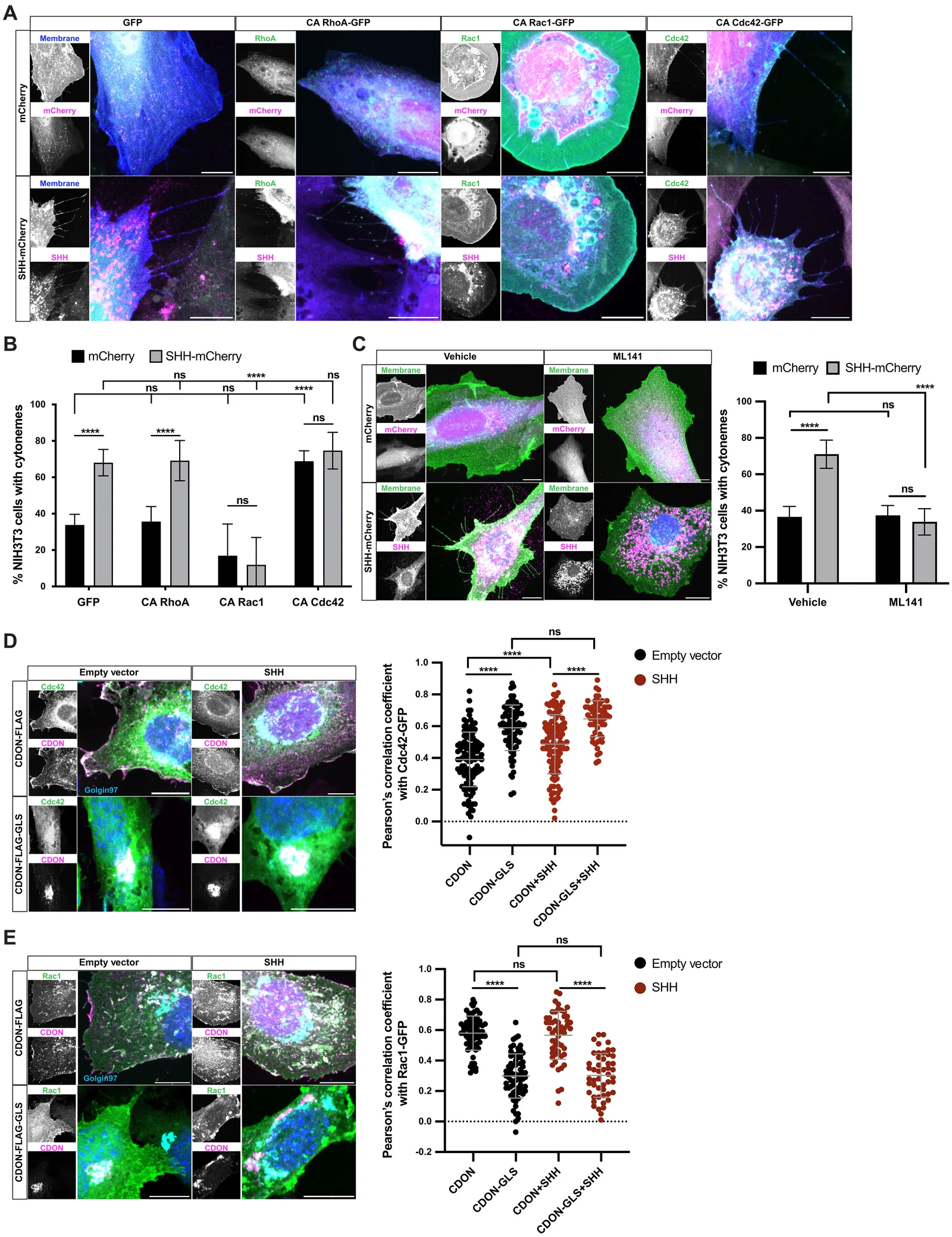
Cdc42 promotes SHH cytoneme formation. (**A**) NIH3T3 cells were transfected with membrane-BFP (blue), mCherry or SHH-mCherry (magenta), and constitutively active (CA) RhoA-GFP, CA Rac1-GFP, or CA Cdc42-GFP (green). (**B**) Cytoneme occurrence rates were quantified from three biological replicates totaling *n* = 239 to 330 cells per condition. (**C**) NIH3T3 cells expressing membrane-pHluorin (green) and mCherry or SHH-mCherry (magenta) were treated with DMSO vehicle or Cdc42 inhibitor ML141. DAPI is shown in blue. Cytoneme occurrence rate was quantified from three biological replicates (*n* = 295 to 384 cells per condition). (**D** and **E**) *Boc*/*Cdon*/*Gas1*^−/−^ MEFs stably expressing MSCV-Hygro (empty vector) or MSCV-Hygro-SHH-FL (SHH) were transfected with WT Cdc42-GFP (D) or WT Rac1-GFP (E) (green) and CDON-FLAG or CDON-FLAG with a Golgi localization sequence (GLS) (magenta). Cells were fixed and stained for Golgin-97 (cyan) and with DAPI (blue). Pearson’s colocalization coefficient was quantified between GFP and anti-FLAG channels within cell body ROIs over two to three biological replicates (*n* = 50 to 109 cells per condition). Scale bars, 10 μm. Data shown as mean ± SD. Significance was calculated using one-way ANOVA. ****P < 0.0001; ns, not significant.

If Cdc42 is a downstream SDC effector that promotes cytoneme formation or elongation, we hypothesized that it is likely recruited to sites of cytoneme outgrowth in a transient manner by CDON or BOC. Thus, we tested whether CDON can recruit Cdc42 to specific subcellular localizations using the GRA as described above. Consistent with CDON being a Cdc42 recruiter, we found that Golgi-localized CDON-GLS relocalized Cdc42-GFP from the cell body to the Golgi (Fig. 6D). Relocalization occurred similarly in both the absence and presence of SHH, suggesting that CDON-mediated Cdc42 recruitment is not ligand dependent (Fig. 6D). Rac1-GFP, which did not influence cytoneme dynamics, was not recruited to the Golgi by CDON-GLS and showed reduced Pearson’s colocalization coefficient values following CDON Golgi tethering (Fig. 6E). These results, along with the results presented above, allowed us to conclude that the SDC components BOC or CDON recruit the GEF FARP1 and its substrate Cdc42 to regulate actin dynamics for cytoneme outgrowth.

### Cdc42 activity in SHH cytonemes requires BOC/CDON

Having identified Cdc42 as the putative GTPase acting downstream of the SDC in cytonemes, we next sought to visualize endogenous Cdc42 activity in the extensions using a Förster resonance energy transfer (FRET)–based Cdc42 biosensor (*95*). A limitation to FRET efficiency analysis is fluorescence intensity–based variation across conditions and samples, which could be exacerbated in thin cytonemes (*96*). To avoid this potential complication, we adapted a fluorescence lifetime imaging microscopy (FLIM)–FRET protocol for quantitative analysis of Cdc42 activity in cell bodies and cytonemes (*96*). We transfected the biosensor into NIH3T3 cells stably expressing SHH-FL and performed live imaging to capture dynamic Cdc42 activity over time (Fig. 7A, fig. S8A, and movie S5). Observation of a singular extension/retraction event demonstrated high Cdc42 activity at the tip during elongation, which was subsequently lost before its retraction toward the cell body (fig. S8A). This behavior is consistent with expected Cdc42 activation dynamics in filopodial-like structures and with the observed colocalization of CDON and FARP1 at cytoneme tips in PLAs, validating that this biosensor is suitable for Cdc42 activity measurements.

**Fig. 7.**
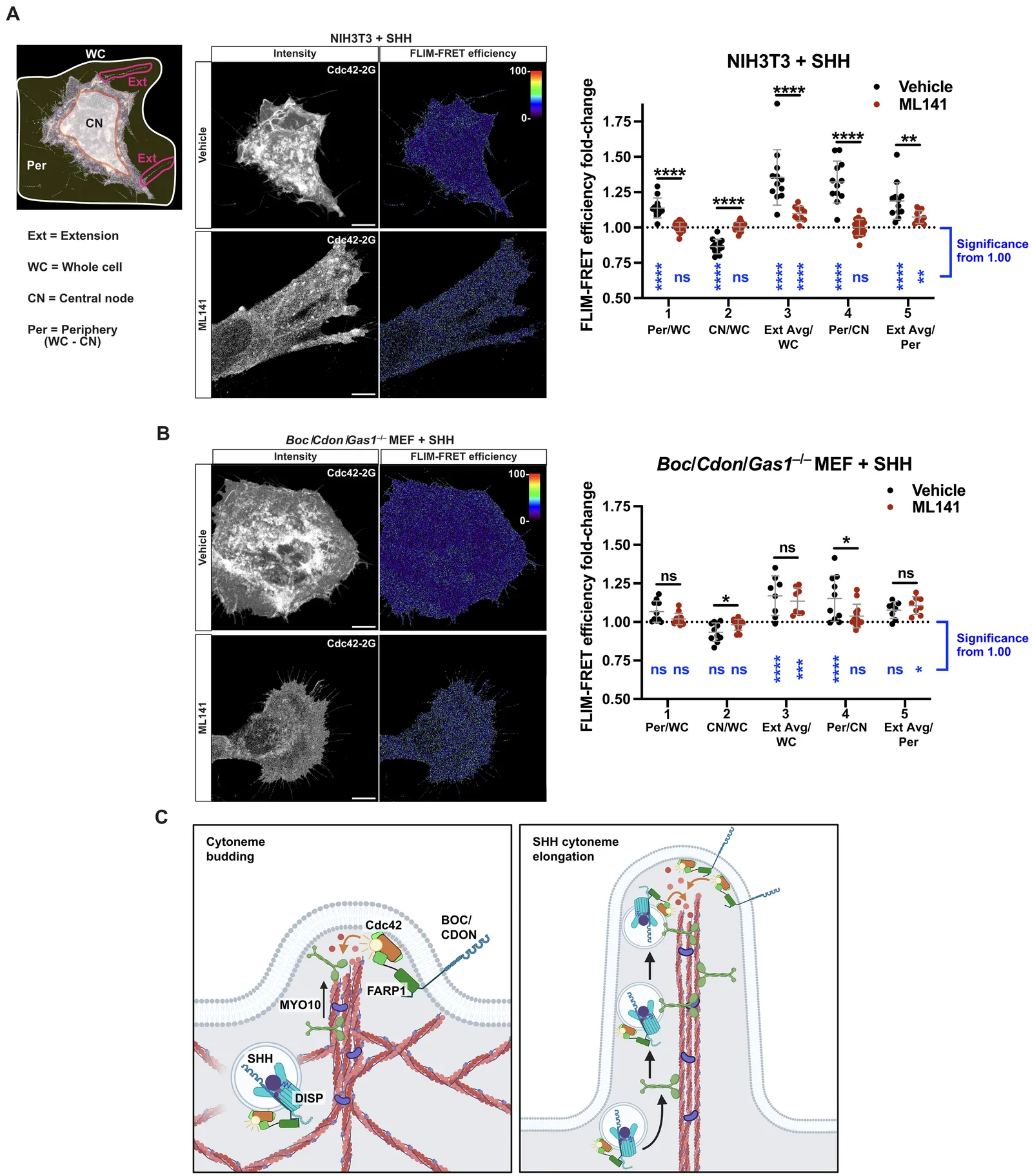
BOC/CDON are required for Cdc42 activity in cytonemes of SHH-expressing cells. (**A** and **B**) A Cdc42-2G Förster resonance energy transfer (FRET)–based biosensor was used to measure endogenous Cdc42 activity. A representative image is shown with Cdc42-2G fluorescent intensity (grayscale) compared to fluorescent lifetime imaging microscopy (FLIM)–FRET efficiency from 0 to 100% (rainbow spectra). Regions of interest (ROIs) were drawn (left) to define the whole cell (WC), ∼5 μm of cell periphery (Per), central node (CN), and cellular extensions (Ext). NIH3T3 cells (A) or *Boc*/*Cdon*/*Gas1*^−/−^ MEFs (B) stably expressing MSCV-Hygro-SHH-FL were transfected with the biosensor, and live imaging was conducted over 15-min time periods for each cell. Cells treated with Cdc42 inhibitor ML141 demonstrate equal subcellular distribution of FLIM-FRET efficiency (bottom panels). Average FLIM-FRET efficiencies were quantified within ROIs and fold change was plotted relative to WC, CN, or Per Cdc42 activity within individual cells as indicated. Cells were treated with DMSO vehicle (black points) or 10 μM ML141 (red points) before imaging. Data are representative of three biological replicates totaling *n* = 10 to 20 cells per condition. Significance was determined from a baseline fold change of 1.0 by one-way ANOVA (blue asterisks). Comparison of vehicle to ML141-treated cells was analyzed by Welch’s *t* test (black asterisks). Scale bars, 10 μm. Data shown as mean ± SD. Significance was calculated using one-way ANOVA. *P < 0.05, **P < 0.01, ***P < 0.001, ****P < 0.0001; ns, not significant. (**C**) A model for co-receptor–mediated cytoneme signaling. CDON or BOC recruits the actin regulators FARP1 and Cdc42 to the plasma membrane. The co-receptors are also components of the DISP and SHH-containing SDC, which is internalized for MYO10-mediated transport down cytoneme lengths. FARP1 and Cdc42 signal to stimulate actin polymerization for cytoneme elongation downstream of CDON or BOC. Created in BioRender. Daly, C. (2026) https://BioRender.com/0jily00.

To quantify activity in distinct subcellular domains, regions of interest (ROIs) including the whole cell (WC), ∼5 μm of the cell periphery (Per), the central node (CN), and individual cellular extensions (Ind Ext) were analyzed (Fig. 7A and fig. S8B). For statistical analysis, individual extension FLIM-FRET efficiency values were averaged to provide a single value per cell for cells that had multiple extensions (Ext Avg versus Ind Ext) (fig. S8B). Because each cell expressed varying baseline Cdc42 biosensor activity, we calculated FLIM-FRET efficiency fold change between ROIs within the same cell to directly compare relative localized Cdc42 activity across multiple cells and experiments (Fig. 7A and fig. S8C). For these assays, significance was calculated against a fold change of 1.00 (Fig. 7A and fig. S8C, blue significance indicators).

Cdc42 activity was elevated at the cell periphery compared to the whole cell or central node (Fig. 7A, Per/WC column 1 and Per/CN column 4, black dots, and fig. S8B). This result was anticipated because Cdc42 requires membrane localization for its activation (*95*, *97*). Consistent with a role for Cdc42 in SHH cytonemes, the highest levels of endogenous GTPase activity were detected in cytoneme-like extensions (Fig. 7A, Ext Avg/WC column 3, black dots, and fig. S8, B and C), even when compared to the cell periphery (Fig. 7A, Ext Avg/Per column 5, black dots, and fig. S8, B and C).

To quantify the magnitude of elevated Cdc42 activity in the extensions, the FLIM-FRET efficiency values for each cell were plotted starting from the central node through the periphery and ultimately to the extension averages (fig. S8B, black solid lines). Least squares regression fits were calculated for each individual cell, resulting in a large average slope value (3195, *R*^2^ = 0.9139), indicating elevated activity in the extensions compared to the other ROIs (fig. S8B, dotted red lines).

To confirm specificity of the active Cdc42 signal, we treated sensor-expressing cells with the Cdc42 inhibitor ML141. ML141 treatment reduced activity of the biosensor, resulting in FLIM-FRET efficiency ratios near baseline and a moderate regression slope from central node to periphery and extensions (589.8, *R*^2^ = 0.9501) (Fig. 7A and fig. S8C, red dots compared to black dots, black asterisks; fig. S8D). On the basis of these results, we conclude that Cdc42 is active in cytonemes of SHH-expressing NIH3T3 cells.

Last, to test whether cytoneme-localized Cdc42 activity in SHH-expressing cells is associated with BOC and/or CDON function, we evaluated biosensor FLIM-FRET efficiency in *Boc*/*Cdon*/*Gas1*^−/−^ MEFs, in which baseline and WNT1-stimulated cytoneme occurrence rates are unchanged and SHH-stimulated cytoneme biogenesis is compromised (*26*) (fig. S2C). Consistent with the inability of SHH to enhance cytoneme occurrence in cells lacking SDC activity, endogenous Cdc42 activity was only modestly enhanced in the *Boc*/*Cdon*/*Gas1*^−/−^ + SHH MEF extensions, resulting in a reduced value for slope from central node through periphery to extensions (1625, *R*^2^ = 0.9223) (fig. S8E). Accordingly, Ext/WC and Ext/Per ratios were decreased (Fig. 7B, columns 3 and 5, black dots; fig. S8F; and movie S6) and ML141 inhibition of Per/WC, Ext/WC, and Ext/Per Cdc42 activity ratios was lost in *Boc*/*Cdon*/*Gas1*^−/−^ cells (Fig. 7B and fig. S8F, red dots compared to black). The ROI regression slope decreased slightly with ML141 treatment (1345, *R*^2^ = 0.8361) (fig. S8G). However, FLIM-FRET efficiency remained comparable to vehicle-treated cells (fig. S8G), suggesting that in the absence of BOC and CDON, Cdc42 activity in cellular extensions of SHH-expressing cells is compromised. Altogether, these results provide supportive evidence that the SDC components BOC and CDON enable localized FARP1-mediated Cdc42 activation for outgrowth of SHH-containing cytonemes in ligand-producing cells (Fig. 7C).

## DISCUSSION

A growing body of evidence supports the idea that cytonemes are key contributors to regulated deployment and reception of intercellular signals during development and tissue homeostasis. They transport morphogens, growth factors, and receptors, and their activity directly promotes signal gradient formation and precision across developing tissues (*10*, *17*–*19*, *24*, *31*, *98*, *99*). Consistent with cytonemes being actin-based structures, their elongation and function are reliant upon actin regulatory proteins (*8*, *18*, *27*, *28*, *31*, *100*). Herein, we demonstrate that, in the case of SHH cytoneme-based transport, BOC and CDON promote activation of the actin regulatory Rho GTPase Cdc42 by recruiting its GEF FARP1. Live imaging of cultured cells reveals that loss of BOC and CDON blunts SHH-stimulated Cdc42 activity in cytonemes, supporting that BOC and CDON confer signaling competence to the SDC for actin control. Live imaging of E9.5 neural tube sections supports that this functionality is conserved in vivo. Deletion of *Cdon* specifically in SHH-producing neuroepithelial cells reduces dynamic cytoneme behavior, occurrence frequency, and length, which correlates with altered ventral cell fate establishment in the developing neural tube. Altogether, these results support a model in which CDON and BOC can function as signaling competent deployment receptors that recruit FARP1 and Cdc42 to instruct actin polymerization for cytoneme elongation and SHH transport (Fig. 7C, left).

Loading of SHH into nascent cytonemes is proposed to occur through entry of SDC-containing vesicles into and along the signaling filopodia through the action of cytoneme-localized MYO10 (*10*, *26*). BOC or CDON-FARP1 signaling from cytoneme membrane and SDC-containing vesicles then ensures continued actin polymerization for cytoneme elongation toward target cells (Fig. 7C, right). Thus, SHH binding proteins of the SDC likely promote ligand transport through linking parallel pathways of (i) DISP-mediated SHH vesicle packaging and (ii) BOC/CDON-mediated cytoneme outgrowth. Whereas DISP is not required for cytoneme elongation, its binding to BOC/CDON spatially couples extension outgrowth with SHH deployment machinery to ensure efficient ligand transport.

BOC and CDON were first identified as founding members of a subfamily of immunoglobulin and fibronectin domain-containing CAM-like proteins that promote myogenesis during skeletal muscle development (*63*, *64*). In this context, CDON signaling leads to activation of Cdc42, which signals to p38/MAPK for muscle differentiation. However, in muscle, Cdc42 recruitment occurs following CDON ligation to N-cadherin, which promotes CDON association with the Cdc42-binding protein Bnip-2 (*65*, *66*, *101*).

Notably, SHH-bound CDON does not associate with Bnip-2 (*101*). Accordingly, BOC and CDON contributions to SHH signaling were initially attributed to them functioning as SHH co-receptors and not to their ability to communicate with actin regulators. Their function in the pathway was thought to be restricted to SHH-receiving cells, where they cooperate with PTCH to enhance SHH signaling during neuronal cell fate specification and axon guidance (*36*, *37*, *83*, *102*). However, PTCH does not require BOC/CDON function for SHH binding (*40*). Moreover, *Boc^−/−^* neural tubes are phenotypically normal at the embryonic stages when progenitor domain establishment occurs, and *Boc^−/−^* mice are viable without overt Hedgehog phenotypes (*51*, *52*). As such, BOC and CDON were proposed to function in the pathway primarily to extend the range of SHH responsiveness to low levels of ligand exposure (*37*). The precise role of CDON in the notochord, which produces SHH but does not respond to it, was not initially clear.

Recent investigations into cytoneme biology provide insight into functions of the co-receptors in signal-producing cells, revealing roles for BOC/CDON and the *Drosophila* CDON ortholog Ihog in cytoneme-based SHH/Hh transport (*14*, *26*, *30*, *33*, *34*, *103*). Like their roles in ligand-receiving cells, cytoneme-specific activity of BOC/CDON in signal-producing cells was initially attributed to activities of their ECDs. Studies in chick limb buds showed BOC and CDON enrichment at cytoneme contact points and, in *Drosophila*, Ihog forms cytoneme-cytoneme connections by associating in trans (*14*, *32*). Ihog can also bind extracellular matrix components to stabilize cytoneme extensions (*17*, *32*, *33*, *103*).

The results presented here suggest that most BOC/CDON cytoneme-promoting activity occurs through their ICDs, which recruit the actin regulators FARP1 and Cdc42. However, CDON mutants lacking an intact ECD cannot promote cytonemes as effectively as the WT protein, suggesting that some cytoneme-promoting activity occurs extracellularly. We speculate that, like the Ihog ECD, the BOC and CDON ECDs could provide adhesion functionality that stabilizes elongated cytonemes, either through homophilic interactions in trans or through binding extracellular interaction partners. Notably, BOC and CDON interact in cis with N-cadherin and M-cadherin at myoblast cell-cell contact sites (*104*), and CDON coordinates N-cadherin positioning at the cell surface for directed neural crest cell migration in zebrafish (*50*). Thus, precedent exists for indirect roles for BOC and CDON in cell-cell adhesion. The contribution of such interactions in a cytoneme context warrants further investigation.

The first direct evidence for BOC or CDON initiating a cellular signal downstream of SHH was revealed through interrogation of BOC function in growth cones of commissural axons. Here, SHH binding to BOC leads to SMO-independent mobilization of the GEFs DOCK3/4, which activate Rac1 for cytoskeletal reorganization (*105*). This observation, combined with the data presented here that both proteins appear to signal in cytonemes for actin regulation, suggests that BOC or CDON recruitment of FARP1 may represent cytoneme-specific signal customization. Notably, FARP1 activity is linked to formation of filopodia and another type of signaling protrusion, the tunneling nanotube (TNT) (*77*, *90*, *106*). Like what is shown here for cytonemes, FARP1-mediated promotion of TNTs requires Cdc42 (*90*). This suggests that Cdc42 recruitment may be a broadly used mechanism by which specialized signaling protrusions are controlled.

Consistent with a conserved role for Cdc42 in cytoneme elongation, in vivo studies demonstrate that its activity is necessary for outgrowth of both WNT8a and NOTCH containing cytonemes. Knockdown of *Cdc42a/c* in zebrafish leads to reduction in numbers and lengths of WNT8a-positive cytonemes, and ML141-mediated Cdc42 inhibition in spinal cord explants reduces cytoneme density across NOTCH-responsive cell populations (*18*, *28*). However, Cdc42-independent cytoneme transport can occur. Cytonemes containing FGFR are dependent on the atypical Rho GTPase RhoD, which signals through mDia3C to stimulate actin polymerization for cytoneme elongation (*27*). Whether this suggests customized engagement of different GEFs/GTPases by distinct signaling pathways or highlights a difference between ligand versus receptor-transporting protrusions is not yet known. However, it is notable that, like BOC and CDON, FGFR contains tandem Ig domains that are essential for its function (*107*). ROR2, which promotes WNT5b and WNT8a cytoneme biogenesis, also harbors Ig domains (*11*, *29*, *108*), suggesting that cargo binding to Ig-containing, adhesion-related molecules may promote cytoneme-based transport.

With respect to both function and regulation, cytonemes appear to share several characteristics with neurons (*109*). Glutamatergic signaling promotes cytoneme formation and stability, and a recent study on WNT7a-containing cytonemes suggests that the signaling protrusions are characteristically similar to dendritic filopodia (*16*, *20*, *110*, *111*). Notably, FARP1 has been demonstrated to promote dynamic behavior of dendritic filopodia in immature neurons and to stimulate formation of vGLUT1/2-positive presynaptic sites in differentiated neurons (*77*, *78*). We propose that BOC and/or CDON recruitment of FARP1 to the SDC redirects FARP1 activity toward cytoneme elongation, highlighting another similarity between neuronal extensions and the specialized signaling filopodia.

Despite their complementary expression domains in the developing neural tube spanning SHH, WNT, and BMP signaling zones, our data suggest that BOC and CDON contributions to cytoneme-mediated signaling may be specific to SHH. *Boc/Cdon/Gas1*^−/−^ MEFs do not show reduced WNT-stimulated cytoneme occurrence rates in vitro. Moreover, the PAX7 expression domain, which is specified by cytoneme cargoes WNT and FGF, does not show overt changes in *Shh-Cre;Boc^−/−^;Cdon^fl/fl^* neural tubes (*8*, *112*, *113*). By contrast, functional loss of the actin motor MYO10 disrupts both ventral SHH activity and dorsal WNT activity in the neural tube, consistent with a broadly used cytoneme function (*10*).

Given that both BOC and CDON can induce cytonemes in cultured cells, and that their cytoneme-promoting activity appears to be specific to SHH, it is tempting to speculate that the split expression domains of *Boc* and *Cdon* in the developing neural tube may directly shape SHH signaling gradient length by promoting cytoneme formation from both signal-sending and -receiving cells. In this scenario, CDON is proposed to function in signal-producing cells of the notochord and floor plate to stimulate extension of dorsally projecting cytonemes for SHH delivery. BOC would then function in signal-receiving cells to induce ventrally projecting cytonemes that synapse with floor plate–derived extensions for ligand delivery along the entire length of the SHH signaling gradient. The proposed activity is supported by studies of the *Drosophila* wing disc that demonstrate that Ptc-containing cytonemes extend from Hh-receiving cells to directly receive ligand from Ihog-induced, signal-sending cytonemes (*31*). Thus, BOC expression in ligand-responding tissue of the neural tube may facilitate dual roles in both cytoneme extension and pathway activation. Clustering of BOC at the plasma membrane through its interaction with PTCH is hypothesized to drive both of these functions.

The proposed model does not exclude the possibility that BOC and CDON could also function in diffusion-based SHH reception. We hypothesize that their activity can be shaped by their subcellular localization such that BOC/CDON localization to cytonemes and/or binding to the SDC promotes communication with actin, and their enrichment on cellular membranes near GAS1 directs them to receive diffusible SHH/SCUBE2 complexes. Consistent with this hypothesis, a recent non–peer-reviewed preprint suggests that Ihog directly binds the *Drosophila* functional ortholog of SCUBE2, Shifted (Shf), to solubilize Hh at cytoneme contact sites (*114*).

The ability of both BOC and CDON to influence cytoskeletal rearrangements in context-dependent manners highlights how functional repurposing of protein activity can be used to customize cellular signaling behavior. The results presented here demonstrate how such customization can ensure proper distribution of SHH from signaling centers like the notochord and floor plate to target cell populations during neuroepithelial cell fate determination. We hypothesize that similar strategies are applied by other signals and receptors transported by cytonemes and suggest that studies aimed at identifying pathway-specific modes of recruiting actin regulators will help to define how signal precision is maintained in dynamic tissue environments.

## MATERIALS AND METHODS

### Animal experiments

Mouse experiments were conducted in accordance with SJCRH/IACUC-approved protocol 608-100616. Animal Research: Reporting In Vivo Experiments guidelines have been followed in method and results reporting. Animal husbandry and procedures adhered to protocols approved by St. Jude Children’s Research Hospital Institutional Animal Care and Use Committee and in accordance with the Guide for the Care and Use of Animals. Rosa26mTmG [B6.129(Cg)-Gt(ROSA)26Sortm4^(ACTB-tdTomato,-EGFP)Luo^/J; JAX no. 007676], Shh-Cre (B6.Cg-Shh^tm1(EGFP/cre)Cjt^/J; JAX no. 005622), and *Boc^−/−^* and *Cdon* flox (*52*–*54*) on a C57BL/6 background were bred to obtain the desired genotypes.

### Generation of cell lines and cell culture

NIH3T3 (CRL-1658) and HEK293T (CRL-11268) cell lines were obtained from ATCC. *Boc*/*Cdon*/*Gas1*^−/−^ MEFs were obtained from B. Allen (*51*). *Disp1*^−/−^ MEFs were obtained from P. Beachy and A. Salic (*42*, *44*). *Ptch1*^−/−^ MEFs (*47*) were obtained from P. Beachy. WT MEFs were published previously (*26*).

The St. Jude Center for Advanced Genome Engineering (CAGE) generated the following: *Disp1/Ptch1/Ptch2^−/−^* MEF, *Boc*/*Disp1*^−/−^ MEF, *Cdon*/*Disp1*^−/−^ MEF, *Boc*/*Cdon*/*Disp1*^−/−^ MEF, and *Boc*/*Cdon*/*Disp1*/*Smo*/*Ptch1*/*Ptch2*^−/−^ C57/BL6 MEF cell lines using CRISPR-Cas9 technology. Briefly, 400,000 *Disp*^−/−^ MEFs (*42*, *44*) were transiently cotransfected with precomplexed ribonuclear proteins (RNPs) consisting of 150 pmol of chemically modified sgRNA (Synthego), 50 pmol of 3× NLS *Sp*Cas9 protein (St. Jude Protein Production Core), and 300 ng of pMaxGFP (Lonza). In MEF lines with multiple genes knocked out, sequential nucleofections were performed to prevent larger chromosomal rearrangements. Transfections were performed at least 7 days apart to allow editing to be completed before targeting the next gene. The transfections were performed via nucleofection (Lonza, 4D-Nucleofector X-unit) using solution P3 and program DS-150 in a small (20 μl) cuvette according to the manufacturer’s recommended protocol. Single cells were sorted based on transfected cells (GFP^+^) 5 days postnucleofection into 96-well plates containing prewarmed media and were clonally expanded. Clones were screened and verified for the desired out-of-frame modifications using targeted deep sequencing analyzed with CRIS.py as previously described (*115*, *116*). Final clones tested negative for mycoplasma by the MycoAlertPlus Mycoplasma Detection Kit (Lonza) and were authenticated using the PowerPlex Fusion System (Promega) performed at the Hartwell Center (St. Jude). Editing construct sequences and screening primers are as follows:

CAGE622.Boc.g6 spacer: UGUGUGGCACGGAUGCCUGC

CAGE622.Boc.F: CTACACGACGCTCTTCCGATCTGTGATCCTGGGCTGTGTGGTGGAGC

CAGE622.Boc.R: CAGACGTGTGCTCTTCCGATCTCCCCCAAGAGTGTTGAGCAGCCACC

CAGE623.Cdon.g3 spacer: GUUGCAUAAUGGAAAACGGU

CAGE623.Cdon.F: CTACACGACGCTCTTCCGATCTGTTCCGTTGTAGACTTGGCA

CAGE623.Cdon.R: CAGACGTGTGCTCTTCCGATCTCAGCACAGAATAGAAAGCTCCTG

SS143.PTCH1.g35 spacer: UGUAGUACACGUGCGUGUGA

SS143.PTCH1.F2: CAGACGTGTGCTCTTCCGATCTTCACAGTAACACCCGGTACA

SS143.PTCH1.R2: CAGACGTGTGCTCTTCCGATCTGGGACTCTGACAGCTGAGGT

CAGE1188.Ptch2.g19 spacer: AUCUGCUGGGACGCGUUGGC

CAGE1188.Ptch2.F: CAGACGTGTGCTCTTCCGATCTCATGGTGCTGCAGGCCTGGCAGAG

CAGE1188.Ptch2.R: CAGACGTGTGCTCTTCCGATCTAGGGAAACTTGCTGGAGGGGAGGCA

CAGE298.Smo.g14 spacer: CAGCUACAUCGCAGCCUUCG

CAGE298.Smo.F: CTACACGACGCTCTTCCGATCTTTCCTTCCCCGTGTCAGAACGAGGT

CAGE298.Smo.R: CAGACGTGTGCTCTTCCGATCTGCGGCCATGCAGTGAAGTGAGGGTC

All cell lines were validated by functional assay such as Western blot, SMO ciliary localization, and/or gene expression analysis when feasible, and tested for mycoplasma contamination once a month using MycoAlert Mycoplasma Detection Kit (Lonza). Cell lines were maintained at 37°C in 5% CO_2_. Complete DMEM (cDMEM) was made by supplementing DMEM (Gibco) with 0.1 mM nonessential amino acids (NEAA), 2 mM l-glutamine, 1% pen-strep, and 1 mM sodium pyruvate. NIH3T3 cell lines were cultured in cDMEM +10% bovine calf serum (BCS, Gibco), while HEK293T and MEF cell lines were cultured in cDMEM +10% fetal bovine serum (FBS, Thermo Fisher Scientific).

### Plasmid constructs/cloning

The following plasmids were used in this study: pcDNA3-EGFP (Addgene no. 13031), pCMV-mCherry-membrane (Addgene no. 55779) (*117*), pCMV-mCherry2, pcDNA3-mouse (m) SHH-FL-mCherry2, hereafter referred to as SHH-FL or SHH (*26*), pcDNA3-SHH-FL-EGFP (*26*), pcDNA3-V5-mDisp1-HA, hereafter referred to as V5-DISP-HA or DISP (*118*), pCS2-human (h) BOC-EGFP and pCS2-hCDON-EGFP, hereafter referred to as BOC or CDON (a gift from A. Salic) (*38*), pcDNA3-FLAG-mTOR (Addgene no. 26603) (*119*), pCig2-GPI-pHluorin (*120*) and pCig2-GPI-BFP (*121*) (gifts from D. Solecki), pCAG-Bgh-GFP-FARP1 (Addgene no. 115501) (*77*), pEGFP-ITSN1-mCherry (Addgene no. 129614) (*122*), pCAGGS-FLAG-DOCK7 (a gift from L. Van Aelst) (*123*), pcDNA3-EGFP-RhoA-Q63L (Addgene no. 12968) (*124*), pcDNA3-EGFP-Rac1-Q61L (Addgene no. 12981) (*124*), pcDNA3-EGFP-Cdc42-Q61L (Addgene no. 12986) (*124*), pcDNA2-EGFP-Rac1 (Addgene no. 12980) (*124*), pTriEx4-Cdc42-2G (Addgene no. 68814) (*95*), pCig2-LifeAct-mTFP1 (a gift from D. Solecki) (*125*), and pCMV-human GLI2-3X-FLAG (Addgene no. 84920).

pCS2-BOC-GFP intracellular domain deletion was accomplished using the QuikChange XL II kit (Agilent). A FLAG tag with stop codon was added to pCS2-CDON-GFP using QuikChange XL II. pCS2-CDON-FLAG domain deletions were accomplished using the In-Fusion cloning kit (Takara). pCS2-CDON-GFP domain deletion constructs were generated from FLAG-tagged constructs through In-Fusion deletion of the FLAG tag and stop codon.

TurboID tag was added through amplification with the addition of restriction digest sites (BOC-FLAG: Spe I; CDON-FLAG: Sma I) and then standard restriction digest cloning (New England Biolabs). WT Cdc42-GFP was cloned from pcDNA3-EGFP-Cdc42-T17N using QuikChange XL II kit (Agilent). DOCK7 was cloned into the pCAG-Bgh-GFP vector using the In-Fusion cloning kit (Takara). GLSs were added through addition of an Eco RI site (QuikChange XL II), amplification of the GLS from mTOR, and subsequent restriction digest cloning. GLI2-Cterm-GFP was cloned from pCMV-hGLI2-FLAG using QuikChange XL II to add a Kozak sequence and then remove the N-terminal domain and the Zinc Fingers. An Emerald-GFP (EmGFP) tag was amplified from pcDNA6.2-EmGFP by Gateway technology (Life Technologies) and was added to GLI2 using Xba I restriction digest cloning. Primer sequences were as follows (Integrated DNA Technologies):

CDON ΔIg F: ACTTGGCAGTTGTTCCTTTTGAAACAAATACAA

CDON ΔIg R: GAACAACTGCCAAGTCTGAACTCACAGA

CDON ΔFN F: TTCCTGATCGTGTTCCTGGAGCTTCTG

CDON ΔFN R: GAACACGATCAGGAACAGAGATGCCGC

CDON ΔTM F: GCAGTGACGCAATGTGCCTGTGGAAGAATCG

CDON ΔTM R: ACATTGCGTCACTGCTTCTGGCAGGG

CDON ΔICD F: TTTTCATTTCTAGTGGTGGAGGCTCTGGG

CDON ΔICD R: CACTAGAAATGAAAACCATCAGAATGAGGACC

CDON ΔFLAG F: GAGGTGGCGTGAGCAAGGGCGAGGAGC

CDON ΔFLAG R: TGCTCACGCCACCTCCACTGCCTCC

BOC ΔICD F: CTTCATCCCCTTCTGCACTAGTGGTGGAGGCT

BOC ΔICD R: AGCCTCCACCACTAGTGCAGAAGGGGATGAAG

TurboID for BOC (Spe I) F: TATAGATAACTAGTGATTACAAGGATG

TurboID for BOC (Spe I) R: TATATATCTACTAGTCTATAGTTCTAGA

TurboID for CDON (Sma I) F: TATATATATCCCGGGAAACCGATTACAAGGATG

TurboID for CDON (Sma I) R: TATATTATACCCGGGCTATAGTTCTAGA

WT Cdc42 F: GGTGCTGTTGGTAAAACTTGTCTCCTGATATCC

WT Cdc42 R: GGATATCAGGAGACAAGTTTTACCAACAGCACC

pCAG-Bgh-GFP vector F: AGCTAGCGCAAGGGCGAATTCTGCA

pCAG-Bgh-GFP vector R: CCTTGCGCTAGCTCCCTTGTACAGCTC

DOCK7 insert F: GGAGCTAGCGCAAGGATGGCCGAGCGCCGCGCCTTC

DOCK7 insert R: GCCCTTGCGCTAGCTTTAGAGATCCATTTTGCGAAGGCTC

mTOR GLS F: TATAACTACGAATTCACTAGTGAAATGCTGGTC

mTOR GLS R: TATAGAATTCTACTACCTATCTCATGAGGGTGAC

Boc/Cdon Add FLAG–Eco RI site F: GTGGAGGTGGCGACTACAAGGATGACGATGACAAGGAATTCAGATCCATGG

Boc/Cdon Add FLAG–Eco RI site R: CCATGGATCTGAATTCCTTGTCATCGTCATCCTTGTAGTCGCCACCTCCAC

Gli2 Add Kozak F: AATTCAGCCACCATGGAGACGTCTGCCTCAGCC

Gli2 Add Kozak R: CTCCATGGTGGCTGAATTCGCGGCCGCAAGC

Gli2 ΔNterm F: GAATTCAGCCACCATGGAGGTGGTCATCTAT

Gli2 ΔNterm R: ATAGATGACCACCTCCATGGTGGCTGAATTC

Gli2 ΔZF F: GCGAATTCAGCCACCATGGAGGGCCCAGATGCCCACGTCAC

Gli2 ΔZF R: GCGAATTCAGCCACCATGGAGGGCCCAGATGCCCACGTCAC

EmGFP F: GACCCAGCTTTCTTGTAC

EmGFP R: TTACTTGTACAGCTCGTCC

CDON ECD mutants (CD4::CDON and ΔECD) were created using the pCS2-CDON-GFP construct through the custom cloning services of GenScript Biotech.

### Plasmid transfection

Cells were seeded at 60 to 80% confluency a day before transfection. Plasmids were transfected using Lipofectamine 3000 (Invitrogen) according to the manufacturer’s protocol. Cells were fixed and/or lysed for analysis ∼24 to 48 hours following transfection.

### siRNA transfection

Cells were reverse transfected using Lipofectamine RNAiMAX (Invitrogen) according to the manufacturer’s protocol. Briefly, 5 μl of RNAiMAX was diluted with either 30 pmol medium GC siScr (Invitrogen 462001) or 10 pmol each of three si*Farp1* Silencer Select siRNAs (Invitrogen 4390771: s104494, s104495, and s104496) in 1 ml of Optimum in 6-well plates and incubated rocking at room temperature (RT) for 10 min. NIH3T3 cells were added in 1 ml of cDMEM at 50% confluency and incubated overnight. The following day, medium was removed for subsequent transfection with plasmids.

### Drug treatments

For Cdc42 inhibition, cells were treated with 10 μM ML141 (Selleck Chemicals S7686) dissolved in DMSO or DMSO vehicle in serum-free DMEM for 1 hour at 37°C before fixation as in (*94*).

For treatment with Smoothened Agonist (SAG), cells were seeded at 90 to 100% confluency. The next day, they were serum starved in cDMEM for 2 hours at 37°C, and then treated with DMSO vehicle or 100 nM SAG (Selleck Chemical Company) diluted in cDMEM + 0.5% FBS overnight at 37°C. Following 24 hours of SAG treatment, the cells were analyzed through qRT-PCR, immunoblotting, or immunofluorescence as described below.

### Quantitative real-time PCR

Cells were seeded into 60- or 100-mm dishes and, if applicable, treated as indicated. Approximately 18 hours postseeding or treatment, total RNA was extracted using TRIzol/chloroform and isopropanol. Then, 1000 ng of RNA was converted into complementary DNA (cDNA) using a High-Capacity cDNA Reverse Transcription kit (Applied Biosystems). qRT-PCR reactions were conducted using a QuantStudio 7 Flex PCR machine using PowerUp Sybr Green Master Mix (Applied Biosystems). Fold change in expression levels was calculated using the ΔΔCt method relative to the *Btf3* housekeeping gene. CRISPR-edited genes were evaluated using primers designed to bind to sgRNA-targeted sequence.

Primer sequences were as follows (IDT):

Boc F: GGATGCCTGCAGGAGCT

Boc R: CCTCGTCTACTTCAATCACATGC

Cdon F: GGAAAACGGTTGGACAGAAAC

Cdon R: GGGAAGGGTTAAGAGACAGAAT

Ptch1 F: CCTCACACGCACGTGTACTA

Ptch 1 R: GGAGTCTGAGAACTGGGAGA

Ptch2 F: CAACGCGTCCCAGCAGAT

Ptch2 R: GCATAGGCCAGCATAAGCAGATAG

Smo F: GCTACATCGCAGCCTTCG

Smo R: GAAGAGAATAACCGCAGGGTAG

Gli1 F: GGTGCTGCCTATAGCCAGTGTCCTC

Gli1 R: GTGCCAATCCGGTGGAGTCAGACCC

Btf3 F: GAACAACATCTCTGGTATTGAAGA

Btf3 R: AATGGTGAAGGTGTTTGCTG

### Embryo vibratome sectioning

Embryos were prepared for cytoneme preservation in a protocol modified from (*9*). Pregnant dams were harvested, uterine horns were removed, and E9.5/25 to 30 somite stage embryos were dissected in phenol-free cDMEM +10% BCS and rinsed in 1× PBS. *Shh-Cre*;*mTmG* embryos were identified using an EVOS M5000, and then embedded in 0.5% sucrose and 4% low–melting point agarose (Invitrogen 16520-050) in 1× PBS with Ca^2+^ and Mg^2+^. Embedded embryos were sectioned using a Leica vibratome VT1200S at 150 μm thickness in cold 1× PBS. For live imaging, sections were incubated in cDMEM +10% BCS in glass bottom 35-mm dishes (MatTek P35G-1.5-14-C) precoated with poly-d-lysine (∼20 μg/ml; Gibco) at 37°C for ∼1 to 2 hours until live imaging. Imaging was conducted on a Nikon A1R (20× objective, pinhole 2.0 AU, Z-stack, two to four line averaging) using a stage-top incubator. For fixed imaging, sections were incubated in 4% paraformaldehyde at RT for 45 min, washed three times for 10 min in 0.1% Triton X-100/1× PBS, rocking at RT. Sections were then blocked rocking at RT for 1 hour or overnight at 4°C in 5% donkey serum + 0.1% Triton X-100 in 1× PBS. Block was replaced with 1:1000 primary antibody diluted in 5% donkey serum + 0.1% Tween-20 in 1× PBS and incubated overnight at 4°C (Goat anti-BOC, R&D Systems AF2385; Goat anti-CDON, R&D Systems AF2429). Sections were washed three times, 10 min, rocking at RT in 0.1% Tween-20 in 1× PBS. Sections were stained in secondary antibody [1:500 Donkey anti-Goat Alexa Fluor 647 (Invitrogen) and 1:2500 DAPI in 5% donkey serum + 0.1% Tween-20 in 1× PBS] for 1.5 hours rocking at RT. Following three 10-min washes in 0.1% Tween-20 in 1× PBS, sections were rinsed in Ultrapure H_2_O and mounted using ProLong Diamond (Thermo Fisher Scientific). Neural tubes were imaged using a Nikon A1R, 20× objective for full neural tube and 60× oil immersion objective for floor plate/notochord zooms.

### Embryo cryosectioning

Frozen embryos were processed for sectioning at E8.5/8 to 15 somites, E9.5/25 to 30 somites, or E10.5. Pregnant dams were harvested, uterine horns were removed, and embryos were dissected, rinsed in 1× PBS, and imaged using a stereo microscope (Leica). Embryos were fixed in 4% paraformaldehyde for 1 hour at RT, washed three times in 1× PBS, and transferred to 30% sucrose 4°C overnight to cryoprotect. Then, embryos were frozen in O.C.T compound (Tissue-Tek) on dry ice. Frozen embryos were sectioned transverse at 10-μm thickness on a Leica CM1950 cryostat.

### RNAscope ISH

Fixed-frozen slides were stained following the RNAscope Multiplex manual assay as described by the manufacturer (Advanced Cell Diagnostics Bio) with the following modifications to preserve tissue integrity: no antigen retrieval step and protease incubation for 10 min. Signal was developed using Opal 620, Opal 650, and Opal 690 fluorophores (Akoya Biosciences) at a 1:1000 dilution. DAPI (Sigma-Aldrich) was added after signal development. Stained sections were mounted in ProLong Gold (Thermo Fisher Scientific). Images were acquired with a Leica Stellaris confocal microscope using a 63× oil objective. Probes: MmCdon-C2 (ACDBio no. 1581041), MmBoc-C3 (ACDBio no. 876211), and MmShh-C4 (ACDBio no. 314361)

### Progenitor domain IHC

Cryosections were dried, washed in 1× TBST, and blocked with 2% BSA + 1% goat serum + 0.1% Triton X-100 in 1× PBS (blocking buffer). Sections were incubated with primary antibodies in blocking buffer overnight at RT in a humidified chamber: mouse anti-SHH (1:10, Developmental Studies Hybridoma Bank 5E1), rabbit anti-FOXA2 (1:300, Abcam ab108422), mouse anti-PAX6 (1:10, Developmental Studies Hybridoma Bank PAX6), rabbit anti-OLIG2 (1:300, Millipore AB9610), rabbit anti-NKX2.2 (1:300, Novus Bio NBP1-82554), and mouse anti-PAX7 (1:10, Developmental Studies Hybridoma Bank PAX7). Sections were washed three times in 1× TBST and then stained with 1:1000 secondary antibodies [Goat α-Mouse Alexa Fluor 488 IgG1γ1 and Goat α-Rabbit Alexa Fluor 555 IgG (H + L), Invitrogen] in blocking buffer for 3 hours at RT. Sections were washed three times in 1× TBST, rinsed in water, and mounted using ProLong Gold mounting media (Thermo Fisher Scientific). Sections were imaged using a Leica DMi8 widefield microscope with a 10× (E10.5) or 20× (E9.5) objective. A minimum of five cardiac-level sections from at least three embryos per genotype were processed to generate representative images. Somite-matched embryos were directly compared.

### Immunocytochemistry and imaging

Cell fixation and staining for cytoneme visualization were performed as previously described using modified electron microscopy (MEM)–fixation protocols (*48*). Cytoneme occurrence rates per coverslip were obtained by quantifying percentage of cells with at least one cytoneme (≥10 μm length) (*26*). Coverslips for GRA and primary cilia immunofluorescence were processed by washing three times with 1× PBS and fixing for 10 min in 4% paraformaldehyde in PBS. Coverslips were then washed three times, 5 min each in 1× PBS, and blocked using 5% donkey serum + 0.1% Triton X-100 in PBS for 1 hour at RT. Primary antibodies were diluted in 5% donkey serum +0.1% Tween-20 in PBS (Wash Buffer) and incubated overnight at 4°C in a humidified chamber. Coverslips were washed with wash buffer 3× for 5 min each at RT and secondary antibodies were added at 1:1000 plus 1:5000 DAPI in wash buffer for 1 hour at RT. Coverslips were washed in wash buffer 3× for 5 min each at RT, rinsed in UltraPure H_2_O, and then mounted using ProLong Glass (Thermo Fisher Scientific). Staining was carried out using the following primary antibodies: Rabbit α-Golgin-97 [1:200, Cell Signaling Technology (D8P2K) 13192], Mouse α-FLAG [1:2000, Sigma-Aldrich (M2) F1804], Rat α-HA [1:300, Roche (3F10) 11867423001], Rabbit α-FARP1 (1:200, Abcam ab236948), Mouse α-SMO [1:500, Santa Cruz Biotechnology (E-5) sc-166685], and Rat α-Arl13b (1:1000, BiCell Scientific 90413); and the following secondary antibodies: Donkey α-Rabbit Alexa Fluor 647, Donkey α-Mouse Alexa Fluor 555, Donkey α-Rat Alexa Fluor 488, and Streptavidin Alexa Fluor 647 (Invitrogen). Microscopy was conducted using a Leica SP8 (63× oil objective) or Nikon A1R (60× oil objective) confocal instrument.

### Proximity ligation assay

PLA was performed using Duolink In Situ Red Kit Mouse/Rabbit (Sigma-Aldrich DUO92101) according to the manufacturer’s protocols. Cells were transfected and seeded on coverslips as described above, and then fixed using the MEM-fixation protocol (*48*). Cells were then blocked and labeled using the Duolink protocol using the following primary antibodies: Rabbit α-CDON (1:2000, ProteinTech 17634-1-AP) and Mouse α-GFP [1:2000, Rockland (9F9.F9) 600-301-215M].

### Image processing and analysis

Following acquisition, images/movies were processed using FIJI/ImageJ (*126*). Immunocytochemistry images are presented as maximum intensity projections of Z-stack acquisition spanning the cells. Images are representative of cells from three biological replicates.

For embryo section live imaging, Z drift was first corrected based on the GFP channel using the Correct 3D Drift stack registration plugin (*127*). Then, maximum intensity projections of ∼5 to 10 Z-stacks were generated to visualize discrete extensions. Movies were exported at 7 frames per second.

Extension quantification was conducted by counting individual membrane-GFP extensions observed over the first 10 min of imaging. One to six sections were imaged per embryo, and total extensions per section were averaged to generate a single plot point per embryo. Four to six embryos per genotype were derived from three *Shh-Cre*;*mTmG* litters and five *Shh-Cre*;*mTmG*; *Boc* and *Cdon* mutant litters and were processed and analyzed. Extension lengths were measured using the FIJI/ImageJ segmented line tool at the time of maximum length (throughout the 10-min imaging period), resulting in 34 to 183 extensions measured per genotype. Quantifications were conducted in a blinded manner, before genotyping by Transnetyx.

Neural tube progenitor domain quantification was conducted using the Polygon selection tool in FIJI/ImageJ. Areas of expression domain were normalized to total neural tube area (area of full neural tube minus area of neural tube lumen) for each image. Perimeter of full neural tube was measured for circumference. The DV axis was measured for FOXA2/SHH sections using the line selection tool drawn from the base of the floor plate to the top of the roof plate. The AB axis was measured for NKX2.2/PAX7 sections using the line selection tool drawn from the neural tube lumen to the exterior limit of the neural tube, along the ventral-most boundary of PAX7 stain.

### Colocalization analysis

Colocalization analysis was carried out using FIJI/ImageJ. Maximum intensity projections were generated, and ROIs were created to outline each cell body individually. Pearson’s correlation coefficient was measured using FIJI coloc2 pipeline between pixels of different fluorescent channels within the cell body ROI.

### Immunoblotting and immunoprecipitation

For immunoblotting, dishes were washed 1× in cold 1× PBS and lysed in a RIPA buffer [1× RIPA (Millipore), 1× protease inhibitor cocktail (Pierce), 1 mM PMSF, and 1 mM sodium orthovanadate]. Lysates were incubated on ice for 15 min and sheared using a 22G needle. Debris was pelleted at 12,000*g* for 20 min at 4°C, protein concentration was normalized through BCA assay, and samples were prepared using 1× SSB (2% SDS, 4% v/v glycerol, 40 mM tris-HCl, pH 6.8, 0.01% bromophenol blue, 2 mM dithiothreitol (DTT), and 2% 2-mercaptoethanol).

For coimmunoprecipitation experiments, 10-cm dishes of cells transfected as described above were washed 1× in cold 1× PBS and lysed in coimmunoprecipitation buffer [0.25% NP40, 20 mM tris-HCl, pH 7.4, 50 mM NaCl, 5% glycerol, 1× protease inhibitor cocktail (Pierce), 1 mM EDTA, 2 mM MgCl_2_, 1 mM PMSF, 1 mM sodium orthovanadate, TURBO DNase (1.5 U/ml; Invitrogen)]. Lysates were incubated on ice for 15 min before clarifying at 12,000*g* for 20 min at 4°C. Lysates were precleared with 30 μl of equilibrated protein A/G plus agarose (Santa Cruz Biotechnology) for 30 min, rotating at 4°C, and protein concentration was normalized through BCA assay. Input samples were prepared for immunoblotting using 1× SSB (2% SDS, 4% v/v glycerol, 40 mM tris-HCl, pH 6.8, 0.01% bromophenol blue, 2 mM DTT, and 2% 2-mercaptoethanol).

Equilibrated agarose beads [EZView Red α-HA Affinity Gel (Sigma-Aldrich) and α-Mouse IgG Agarose (Sigma-Aldrich)] were blocked in 1% BSA in TBS for 30 min, rotating, at 4°C, and then washed in TBS followed by immunoprecipitation buffer. Normalized lysates (∼1 to 2 mg) were rotated with 30 μl of agarose at 4°C for 1.5 hours. Beads were washed three times, 5 min each, in 1 ml of immunoprecipitation buffer, before elution for 30 min at RT in 2.5× SSB.

SDS-PAGE samples were run on 4 to 15% TGX gels (Bio-Rad) and transferred onto methanol-activated Immobilon-P PVDF membranes (Millipore) using tris/glycine buffer (Bio-Rad) containing 10% methanol at 100 V for 30 min. Membranes were blocked with 5% milk in tris-buffered saline with 0.1% Tween-20 (TBST) before blotting with appropriate primary antibodies diluted in blocking buffer overnight rocking at 4°C. Following three 5-min washes in TBST, rocking at RT, blots were incubated with HRP-conjugated secondary antibodies (Jackson ImmunoResearch) diluted at 1:5000 in 1.25% milk in TBST for 1 hour, rocking at RT. Blots were washed three more times for 5 min each in TBST, and then developed using SuperSignal West Pico PLUS ECL (Thermo Fisher Scientific) on a ChemiDoc Imaging System (Bio-Rad).

Primary antibodies used were as follows: Rat α-HA 1:3000 [1:300, Roche (3F10) 11867423001], Mouse α-FLAG M2 1:2000 (Sigma-Aldrich F1804), Rabbit α-GFP 1:8000 (Rockland 600-401-215), Rabbit α-Kif5B 1:5000 (Abcam ab167429), Mouse α-Beta-actin 1:3000 [Santa Cruz Biotechnology (AC-15) sc-69879], Rabbit α-GAPDH 1:1000 [Cell Signaling Technology (D16H11) 5174], Rabbit α-SHH 1:2000 [Cell Signaling Technology (C9C5) 2207], Rabbit α-Lamin B1 1:3000 (Abcam ab16048), Mouse α-alpha-Tubulin 1:5000 [Cell Signaling Technology (DM1A) 3873], Rabbit α-CDON 1:1000 (ProteinTech 17634-1-AP), Mouse α-BirA (TurboID) 1:2000 (Abcam ab232732), Goat α-BOC 1:1000 (R&D Systems AF2385), Mouse α-SMO 1:500 [Santa Cruz Biotechnology (E-5) sc-166685], Mouse α-GLI1 1:1000 [Cell Signaling Technology (L42B10) 2643], Rat α-Arl13b 1:1000 [BiCell Scientific (ARL2L1) 90413], and Rabbit α-FARP1 (1:1000, Abcam ab236948).

### Cell surface biotinylation

Cells were seeded into 10-cm dishes and transfected as described above. Before biotinylation, cells were washed twice with ice-cold PBS. Cells were incubated in PBS containing EZ-link Sulfo-NHS-SS-Biotin (0.5 mg/ml; Thermo Fisher Scientific 21331) and incubated at RT for 30 min. The biotinylation reaction was quenched with two 10-min washes in 100 mM glycine in PBS, rocking on ice. Cells were lysed in 1× RIPA + 0.1% SDS + 1 mM PMSF + 2 mM sodium orthovanadate + 1× protease inhibitor cocktail (Pierce). Lysates were needled, incubated on ice for 15 min, and then clarified at 12,000*g* for 20 min at 4°C. Input samples were collected and mixed with 5× SSB for SDS-PAGE. The remaining lysate was subject to incubation with 40 μl of streptavidin-conjugated magnetic beads (Thermo Fisher Scientific) per sample for 2 hours, rotating, at 4°C. Beads were washed three times for 5 min at RT in lysis buffer and then proteins were eluted at RT for 30 min in 2× SSB with 2 mM biotin. Blots are representative of a minimum of two biological replicates.

### Densitometry calculation

Cell surface fraction was quantified through densitometry measurements on FIJI/ImageJ. Densitometry of biotinylated fraction was divided by densitometry of input fraction. Fold change was calculated based on reference protein (WT, represented as 1.0).

### Glycosylation analysis

Glycosylation analysis was performed as previously described (*128*). Cells were lysed in lysis buffer (1× RIPA + 1× protease inhibitor cocktail + 0.8 mM DTT + 0.1% SDS + 1 mM PMSF +2 mM sodium orthovanadate) and cleared at 12,000*g* for 20 min at 4°C. Supernatants were mixed with NEB Buffers 2 or 3, as appropriate, and treated with 1000 U peptide-*N*-glycosidase F (PNGase), 1000 U endoglycosidase H (EndoH), or 800 U *O*-glycosidase + 100 U Neuraminidase (New England Biolabs) for 1.5 hours at RT followed by immunoblotting analysis. Blots are representative of a minimum of two biological replicates. N-linked glycosylation was predicted using the NetNGlyc Server (*129*).

### Biosensor FRET/FRET-FLIM

NIH3T3 cells and *Boc*/*Cdon*/*Gas1*^−/−^ MEFs stably expressing SHH were transfected with LifeAct-mTFP1 (*125*) or Cdc42-2G (*95*) FRET sensor as described above. The following day, cells were seeded into eight-well high glass bottom slides (Ibidi 80807-120) and allowed to recover for several hours. Before imaging, medium was replaced with serum-free, phenol-free medium supplemented with vehicle (DMSO) or Cdc42 inhibitor ML141 (10 μM) dissolved in DMSO.

Live-cell imaging and FRET/FLIM analysis was performed on a Stellaris 8 tauSTED with LAS X FLIM/FCS capabilities (v4.7, Leica Microsystems). mTFP1 signal was acquired with a white light laser tuned to 458 nm with detector range set to 470 to 547 nm in photon counting mode and through a 63× 1.4 NA oil immersion objective. Individual cell volumes were acquired over four to five *Z* steps to image the volume of the cell body for 15 min. The two sequential *Z* planes covering the most extensions and cell body were binned together to ensure a minimum of 700,000 photons acquired per time point series for accurate FLIM-FRET efficiency analysis within cytonemes, extensions, and the cell body. Under these conditions, mTFP1 fluorescent lifetime was determined to be 2.656 ± 0.0199 ns (FLIM fit model: one-exponential reconvolution equation, confirmed by phasor plot), acquired from 5 to 10 LifeAct-mTFP1-expressing cells before each biological replicate. Using this unquenched donor lifetime value, 10 to 20 Cdc42-2G-expressing cells per condition were imaged. FLIM-FRET efficiency was calculated (FRET fit model: mono-exponential donor) using a threshold of 1 to 2 counts and a pixel binning of 2. In the resulting image series, the intensity of the pixels represents 0 to 100% efficiency.

Average FLIM-FRET efficiency for Cdc42-2G time series in various structures of interest were calculated in FIJI/ImageJ. Here, ROIs were outlined as shown in Fig. 7A, defining the whole cell (WC), ∼5-μm-wide periphery of the cell (Per), the central node of the cell (CN), and 0 to 8 individual extensions per cell (Ind Ext), which were averaged to generate a single value per cell (Ext Avg). The thresholding of ROIs was implemented to exclude pixels where no FLIM-FRET was detected (values = 0), allowing exclusion of background and neighboring ROIs. Output FLIM-FRET efficiency values of ROIs were measured across the entire time series and averaged. Raw efficiency values were plotted as CN → Per→Ext Avg to determine regression slopes and *R*^2^ from least squares analysis. Ext Avg efficiency values were compared to WC, CN, and Per efficiency values using Mixed effects analysis. Ratio analysis of ROIs was measured internally across each cell and normalized to account for expression variability of Cdc42-2G, as this can potentially affect FRET-FLIM efficiency values between analyzed cells. Values equal to 1 assume equal distribution of FLIM-FRET efficiency. Ratios significantly < or >1 indicate fold change in activity of Cdc42-2G. Analysis was conducted unblinded. Significance was determined by one-way analysis of variance (ANOVA), with Tukey’s multiple comparison as a posttest. Comparison of vehicle- to ML141-treated cell ratios was analyzed by Welch’s *t* test.

### TurboID-mediated proximity labeling

#### Sample preparation

Proximity labeling was carried out as described previously (*69*). Specifically, 15-cm dishes of *Boc*/*Cdon*/*Disp1*/*Ptch1*/*Ptch2*/*Smo*^−/−^ MEFs expressing TurboID constructs were treated with 100 μM biotin in cDMEM + 10% BCS for 2 hours at 37°C. Cells were lysed and subjected to streptavidin-mediated pulldown (250 μl magnetic beads per sample, Thermo Fisher Scientific). Biotinylated proteins were eluted in sterile-filtered 5× SSB supplemented with 2 mM biotin.

Protein samples were run on a short gel as described in (*130*). The gel bands were destained, reduced with DTT (Sigma-Aldrich), alkylated by iodoacetamide (Sigma-Aldrich), washed, dried down, and rehydrated with a buffer containing trypsin (Promega). After overnight proteolysis at 37°C, peptides were extracted in acetonitrile, dried down in a speed vacuum, and reconstituted in 5% formic acid.

#### Mass spectrometry

The peptide mixture from each band was separated on a nanoscale capillary reverse-phase PepMap RSLC C18 column (75 id, 15 cm) by an HPLC system (Thermo Ultimate 3000). Buffer A was 0.2% formic acid and buffer B was 70% acetonitrile and 0.2% formic acid. The peptides were eluted by increasing organic over a 75-min liquid chromatography gradient. The peptides were ionized by electrospray ionization and detected by a Thermo Orbitrap Exploris 480 mass spectrometer. The mass spectrometer was operated in data-dependent mode. For each 3-s duty cycle, a high-resolution survey scan and several MS/MS scans were acquired in the Orbitrap.

#### Database search and analysis

JUMP (*131*) search engine was used for database searches. The UniProt mouse database was concatenated with a reversed decoy database for evaluating false discovery rate. A mass tolerance of 25 ppm for precursor and product ions was used for database search. Two missed cleavages, maximum of three modifications, were allowed, and assignment of b and y ions was used for identification. Carbamidomethylation of cysteine (+57.02146 Da) for static modification and oxidation of methionine (+15.99492 Da) for dynamic modification were considered. Mass accuracy and matching score filters were used for MS/MS spectra to reduce protein false discovery rate to <1%. Spectral counts of each protein may reflect their relative abundance in the sample after normalizing for protein molecular weight. Common proteomic contaminants were filtered out. The spectral counts between samples for a given protein were used to calculate the *P* value, which is derived by *G* test (*132*).

#### Statistical analyses

Analyses were performed using GraphPad Prism v11.0.2. Ordinary one-way ANOVA was performed for multiple comparisons, comparing the means of each dataset to one another, with Tukey’s multiple comparison as a posttest. Significant differences between two values were determined by two-tailed Student’s *t* tests. Statistics for FLIM-FRET analysis are described above. All quantified data are presented as mean ± SD, with *P* < 0.05 considered statistically significant. Significance is indicated as *P < 0.05, **P < 0.01, ***P < 0.001, ****P < 0.0001; ns, not significant.
