# Supplementary material for: Cell adhesion molecule–related proteins signal to promote cytoneme elongation from Sonic Hedgehog–producing cells
Source: Sci Adv. 2026 Nov 11;12(46):eaei4501. doi: 10.1126/sciadv.aei4501 (PMC13754027; doi:10.1126/sciadv.aei4501)
Supplement: Supplementary file 1 [file sciadv.aei4501_sm.pdf]

Science Advances
AAAS

Supplementary Materials for

Cell adhesion molecule–related proteins signal to promote cytoneme elongation from Sonic Hedgehog–producing cells

Christina A. Daly *et al.*

Corresponding author: Stacey K. Ogden, stacey.ogden@stjude.org

Sci. Adv. **12**, eaei4501 (2026)
DOI: 10.1126/sciadv.aei4501

The PDF file includes:

Figs. S1 to S8
Legends for movies S1 to S6
Legend for data S1

Other Supplementary Material for this manuscript includes the following:

Movies S1 to S6
Data S1

A

	Total	Total indel	indel #1	#1 Reads (%)	indel #2	#2 Reads (%)	indel #3	#3 Reads (%)	indel #4	#4 Reads (%)	indel #5	#5 Reads (%)
*Disp1/**Ptch1**/Ptch2$^{-/-}$*	1143	99.4%	-2	586 (51.3%)	-1	520 (45.5%)	-3	28 (2.4%)				
*Disp1/Ptch1/**Ptch2**$^{-/-}$*	1021	100%	-4	509 (49.9%)	-1	499 (48.9%)	-5	9 (0.9%)				
***Boc**/Disp1$^{-/-}$*	177	98.9%	-1	84 (47.5%)	-13	44 (24.9%)	-2	42 (23.7%)	0	2 (1.1%)		
***Cdon**/Disp1$^{-/-}$*	5308	98.9%	-22	1814 (34.2%)	1	1659 (31.3%)	-4	1634 (30.8%)	-5	64 (1.2%)		
***Boc**/Cdon/Disp1$^{-/-}$*	599	99.5%	-4	224 (37.4%)	-1	137 (22.9%)	1	117 (19.5%)	-2	104 (17.4%)	-5	5 (0.8%)
*Boc/**Cdon**/Disp1$^{-/-}$*	1107	99.7%	-1	682 (61.6%)	-4	393 (35.5%)	-2	16 (1.4%)	-5	11 (1.0%)		
*Boc/Cdon/Disp1/**Ptch1**/Ptch2/Smo$^{-/-}$*	4531	99.7%	-124	3364 (74.2%)	-2	771 (17.0%)	-125	145 (3.2%)	-1	50 (1.1%)	-3	40 (0.9%)
*Boc/Cdon/Disp1/Ptch1/**Ptch2**/Smo$^{-/-}$*	4905	99.3%	-1	2005 (40.9%)	-14	1401 (28.6%)	1	1373 (28.0%)	-2	56 (1.1%)	0	35 (0.7%)
*Boc/Cdon/Disp1/Ptch1/Ptch2/**Smo**$^{-/-}$*	2297	99.1%	-2	707 (30.8%)	-1	520 (22.6%)	-4	518 (22.6%)	1	488 (21.2%)	-3	25 (1.1%)

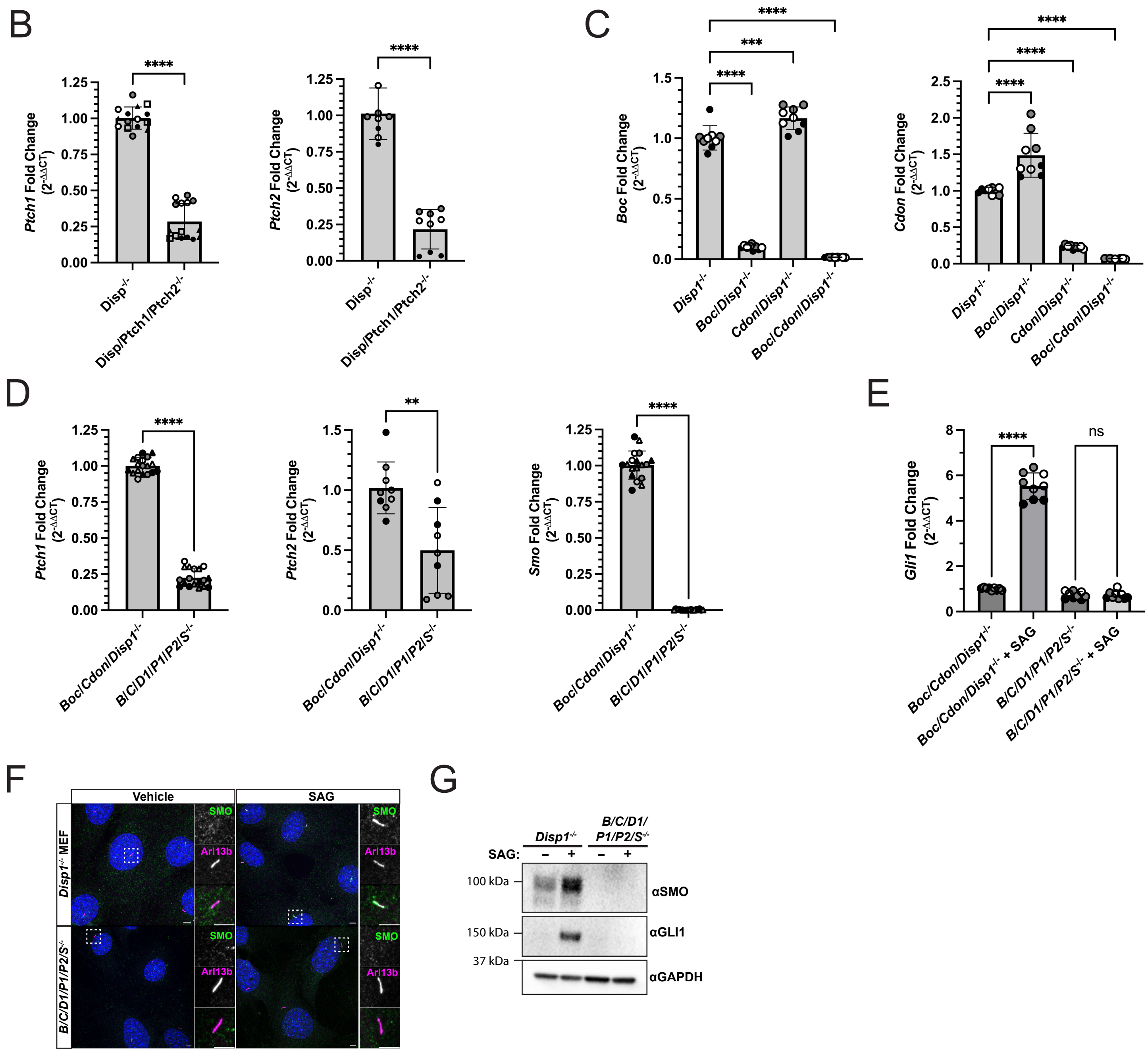

Fig. S1.

(**A**) *Disp1*/*Ptch1*/*Ptch2*$^{-/-}$, *Boc*/*Disp1*$^{-/-}$, *Cdon*/*Disp1*$^{-/-}$, and *Boc*/*Cdon*/*Disp1*$^{-/-}$ Mouse Embryonic Fibroblasts (MEFs) were generated by CRISPR/Cas9 targeting of *Ptch1*, *Ptch2*, *Boc,* and/or *Cdon* in *Disp1*$^{-/-}$ MEFs. *Boc*/*Cdon*/*Disp1*/*Ptch1*/*Ptch2*/*Smo*$^{-/-}$ MEFs were generated by targeting *Ptch1*, *Ptch2*, and *Smo* in the *Boc*/*Cdon*/*Disp1*$^{-/-}$ MEFs. Indels resulting at alleles of targeted genes are shown. (**B**) RT-qPCR validation of *Ptch1* and *Ptch2* knockout compared to *Disp1*$^{-/-}$ MEFs calculated as fold-change ($2^{-\Delta\Delta CT}$) based on *Ppia* reference gene. (**C**) *Boc* and *Cdon* knockout compared to parental *Disp1*$^{-/-}$ MEF line calculated as fold-change ($2^{-\Delta\Delta CT}$) based on *Btf3* reference gene. (**D**) RT-qPCR validation of *Ptch1*, *Ptch2*, and *Smo* knockout as fold-change from *Boc*/*Cdon*/*Disp1*$^{-/-}$ MEFs. (**E**) MEFs were treated for ~18h with DMSO vehicle or Smoothened Agonist (SAG), and SHH pathway induction was measured through RT-qPCR of *Gli1* fold-change based on *Btf3* reference gene. Three to six biological replicates were performed in triplicate (color/shape of graphed points denotes technical replicates), and significance was calculated using one-way ANOVA (**C**,**E**) or student's t-test (**B**,**D**). **$p < 0.01$, ***$p < 0.001$, ****$p < 0.0001$, ns = not significant. (**F**) Immunofluorescence of SMO (green) ciliary translocation following ~18h treatment with DMSO vehicle or Smoothened Agonist (SAG). Arl13b (magenta) serves as a ciliary marker and DAPI is in blue. Scale bars represent 5 µm. (**G**) Immunoblot validation of *Smo* knockout in SAG-treated cells shows that GLI1 expression is no longer induced in *Boc*/*Cdon*/*Disp1*/*Ptch1*/*Ptch2*/*Smo*$^{-/-}$ MEFs. GAPDH serves as loading control. Blot is representative of 3 biological replicates.

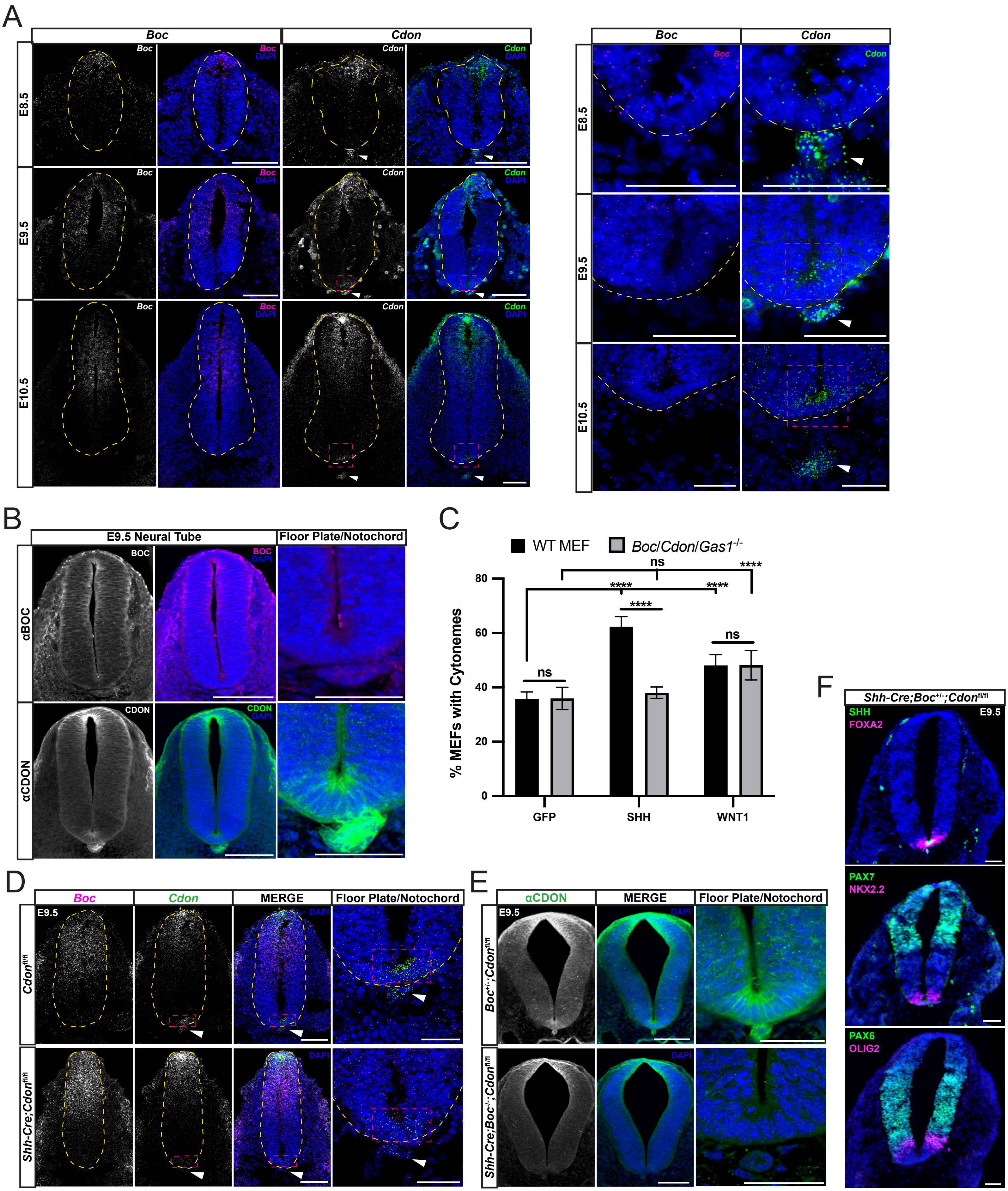
A
Boc
Cdon
E8.5
E9.5
E10.5
DAPI
B
E9.5 Neural Tube
Floor Plate/Notochord
αBOC
αCDON
BOC
CDON
DAPI
C
WT MEF
Boc/Cdon/Gas1-/-
% MEFs with Cytonemes
0
20
40
60
80
ns
****
GFP
SHH
WNT1
D
Boc
Cdon
MERGE
Floor Plate/Notochord
E9.5
Cdonfl/fl
Shh-Cre;Cdonfl/fl
DAPI
E
αCDON
MERGE
Floor Plate/Notochord
E9.5
Boc+/-;Cdonfl/fl
Shh-Cre;Boc+/-;Cdonfl/fl
DAPI
F
Shh-Cre;Boc+/-;Cdonfl/fl
SHH
FOXA2
E9.5
PAX7
NKX2.2
PAX6
OLIG2

Fig. S2.

(**A**) RNAscope *Boc* and *Cdon in situ* hybridization (ISH) in E8.5/10 somites, E9.5/24 somites, and E10.5 embryos. *Boc* probe is magenta, *Cdon* probe is green. Yellow dotted line indicates neural tube, white arrowhead indicates *Cdon* expression in notochord, and magenta box indicates *Cdon* expression in floor plate. (**B**) Representative immunohistochemistry of BOC (magenta) and CDON (green) in E9.5/25 somite embryos with DAPI in blue. (**C**) Cytoneme occurrence rates for WT or *Boc*/*Cdon*/*Gas1*$^{-/-}$ MEFs expressing membrane-mCherry and GFP, SHH-GFP, or WNT1-GFP. Graphs represent three biological replicates totaling n = 195-496 cells per condition. Data shown as mean ± SD. Significance was calculated using One-Way ANOVA. ****$p < 0.0001$, ns = not significant. (**D**) RNAscope *Boc* and *Cdon* ISH in E9.5/24-25 somite neural tubes of the indicated genotypes shows reduction of *Cdon* transcript in floor plate and notochord of *Shh-Cre;Cdon*$^{flox/flox}$ embryos. (**E**) Immunohistochemistry of CDON protein (green) showing localization in E9.5 neural tubes of indicated genotypes to confirm conditional gene deletion reduces protein levels. RNAscope neural tube scale bars = 100 µm and floor plate/notochord scale bars represent 50 µm (**A**,**D**). Neural tube IHC images were taken with a 20X objective, scale bar = 100 µm; Floor plate/notochord IHC images were taken with a 60X objective, scale bar = 50 µm (**B**,**E**). (**F**) E9.5/23-24 somite stage *Shh-Cre;Boc*$^{+/-}$*;Cdon*$^{flox/flox}$ embryo sections were stained for FOXA2 (magenta) and SHH (green), NKX2.2 (magenta) and PAX7 (green), and OLIG2 (magenta) and PAX6 (green). Scale bars = 50 µm. DAPI is in blue for all images.

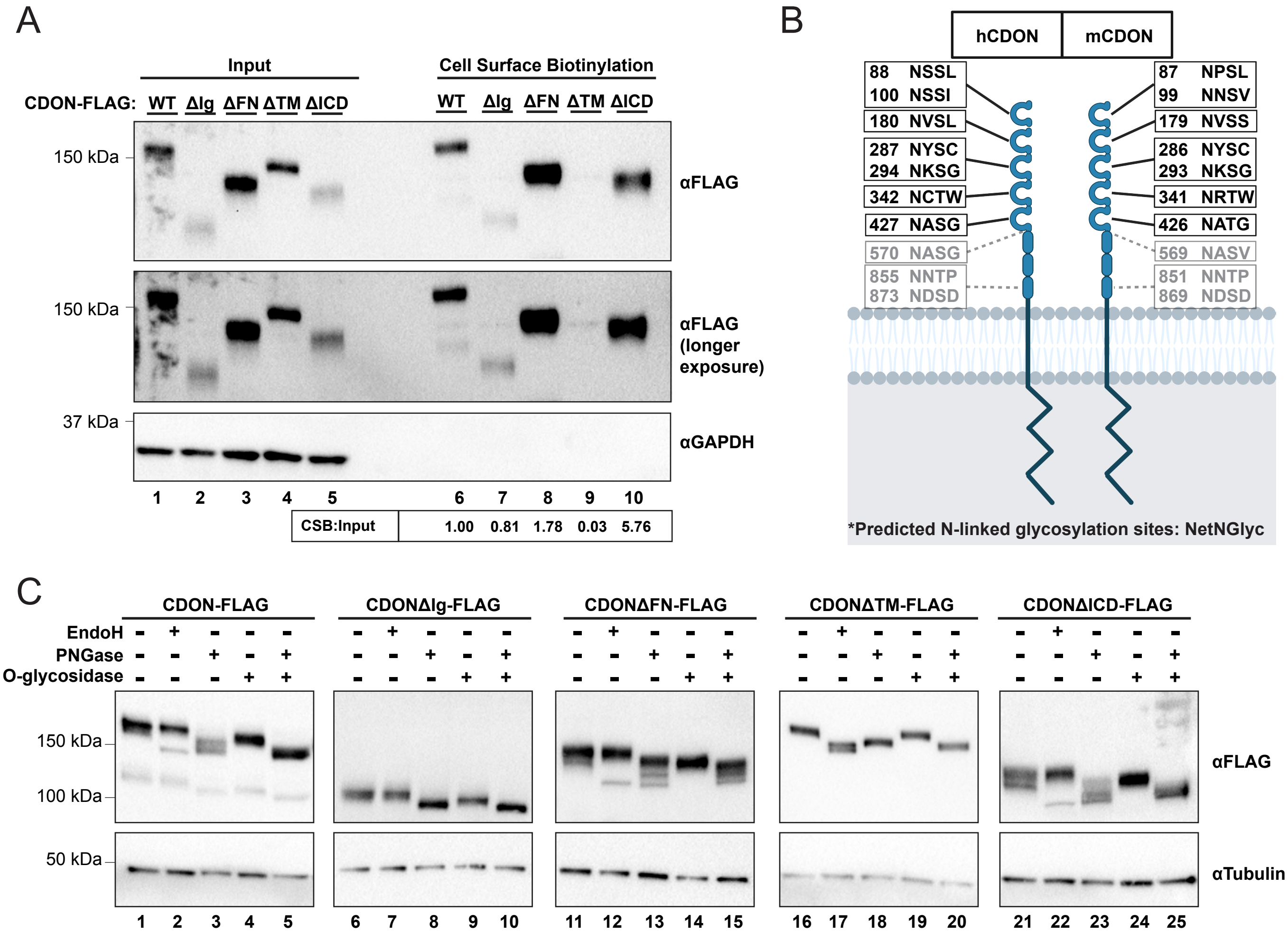

A
Input
Cell Surface Biotinylation
CDON-FLAG:
WT
ΔIg
ΔFN
ΔTM
ΔICD
150 kDa
αFLAG
αFLAG (longer exposure)
37 kDa
αGAPDH
1 2 3 4 5 6 7 8 9 10
CSB:Input
1.00 0.81 1.78 0.03 5.76
B
hCDON
mCDON
88 NSSL
100 NSSI
180 NVSL
287 NYSC
294 NKSG
342 NCTW
427 NASG
570 NASG
855 NNTP
873 NDSD
87 NPSL
99 NNSV
179 NVSS
286 NYSC
293 NKSG
341 NRTW
426 NATG
569 NASV
851 NNTP
869 NDSD
*Predicted N-linked glycosylation sites: NetNGlyc
C
CDON-FLAG
CDONΔIg-FLAG
CDONΔFN-FLAG
CDONΔTM-FLAG
CDONΔICD-FLAG
EndoH
PNGase
O-glycosidase
150 kDa
100 kDa
50 kDa
αFLAG
αTubulin
1 2 3 4 5 6 7 8 9 10 11 12 13 14 15 16 17 18 19 20 21 22 23 24 25

Fig. S3.

(**A**) Cell surface biotinylation (CSB) of CDON-FLAG deletion mutants expressed in NIH3T3 cells. Biotinylated proteins were isolated by Streptavidin-conjugated magnetic beads. GAPDH serves as a loading control. Relative CSB:Input levels were calculated based on the blot shown in the figure. (**B**) NetNGlyc prediction of N-linked glycosylation in human (left) or mouse (right) CDON extracellular domains shows most putative glycosylation sites are in the Ig domains (black vs. gray). Diagram figure created in BioRender. Daly, C. (2026) https://BioRender.com/4q58dct (**C**) NIH3T3 cell lysates expressing CDON-FLAG deletion mutants were treated with EndoH, PNGase, and/or O-glycosidase. Shifts in molecular weight compared to proteins from untreated lysates represent removal of N- and O-linked glycosylation. Tubulin serves as a loading control. Immunoblots are representative of at least 2 biological replicates.

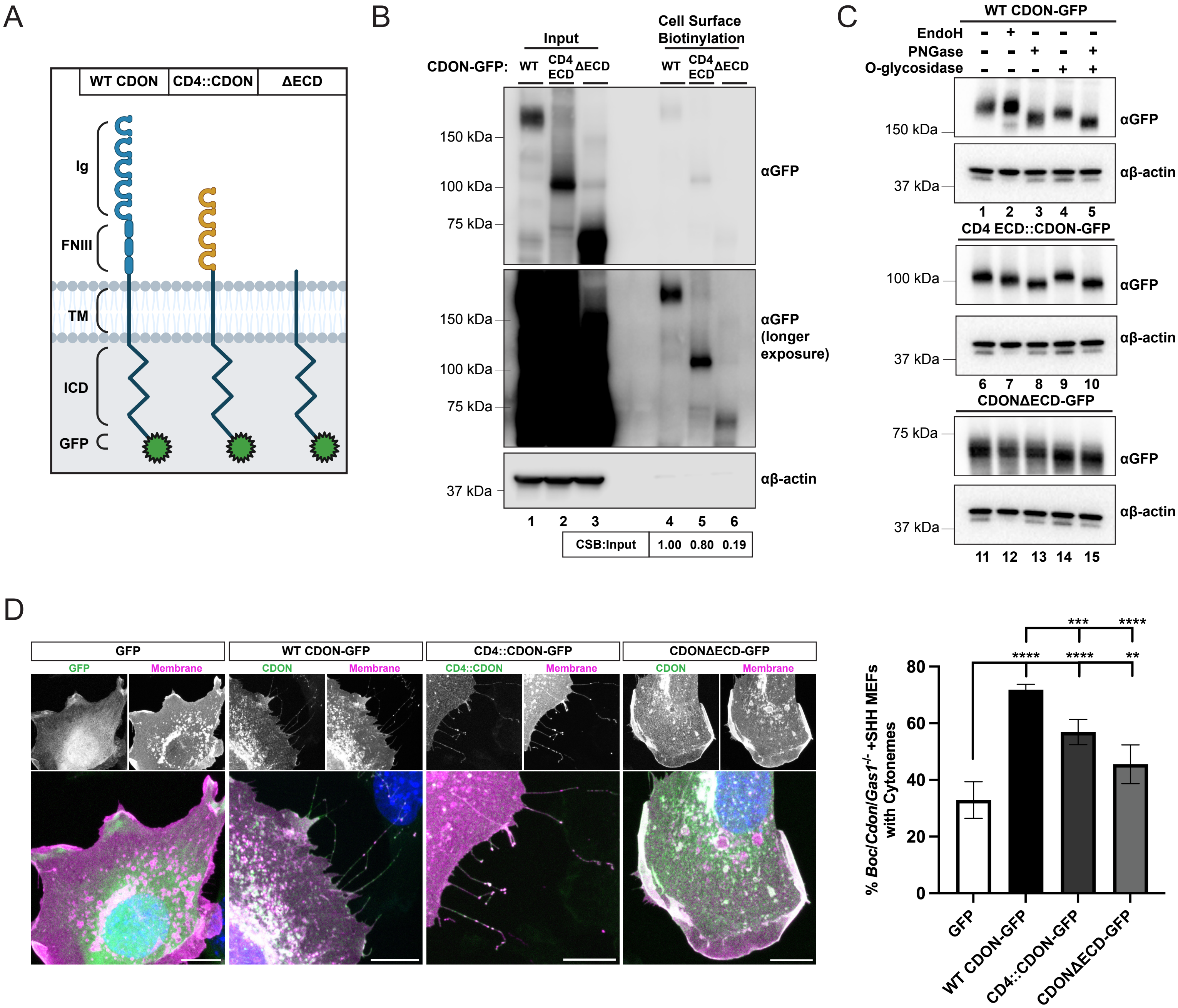

A
WT CDON
CD4::CDON
ΔECD
Ig
FNIII
TM
ICD
GFP
B
Input
Cell Surface Biotinylation
CDON-GFP:
WT
CD4 ECD
ΔECD
150 kDa
100 kDa
75 kDa
αGFP
αGFP (longer exposure)
37 kDa
αβ-actin
1 2 3 4 5 6
CSB:Input
1.00 0.80 0.19
C
WT CDON-GFP
EndoH
PNGase
O-glycosidase
αGFP
αβ-actin
1 2 3 4 5
CD4 ECD::CDON-GFP
6 7 8 9 10
CDONΔECD-GFP
11 12 13 14 15
D
GFP
WT CDON-GFP
CD4::CDON-GFP
CDONΔECD-GFP
CDON
CD4::CDON
Membrane
% Boc/Cdon/Gas1-/- +SHH MEFs with Cytonemes
0
20
40
60
80
****
***
**

Fig. S4.

(**A**) CDON extracellular domain (ECD) mutants were generated such that the CDON ECD was replaced with the ECD of the CD4 receptor (CD4::CDON) or removed (ΔECD). Both constructs retain the CDON signal sequence and the FN-TM linker. Diagram figure created in BioRender. Daly, C. (2026) https://BioRender.com/hperxmj (**B**) Cell surface biotinylation (CSB) of CDON-GFP ECD mutants expressed in NIH3T3 cells. Biotinylated proteins were isolated by Streptavidin-conjugated magnetic beads. β-actin serves as a loading control. Relative CSB:Input ratios were calculated based on the blot shown. (**C**) NIH3T3 cell lysates expressing CDON-GFP ECD mutants were treated with EndoH, PNGase, and/or O-glycosidase. Shifts in molecular weight occur upon removal of N- and O-linked glycosylation. β-actin serves as a loading control. Immunoblots are representative of 2-3 biological replicates. (**D**) *Boc*/*Cdon*/*Gas1*$^{-/-}$ MEFs stably expressing SHH-FL were transfected with membrane-mCherry (magenta) and either GFP, WT CDON-GFP, or the GFP-tagged CDON ECD mutants (green). DAPI is shown in blue. Scale bars = 10 µm. Cytoneme occurrence rates were quantified over three biological replicates (n = 284-378 cells per condition).

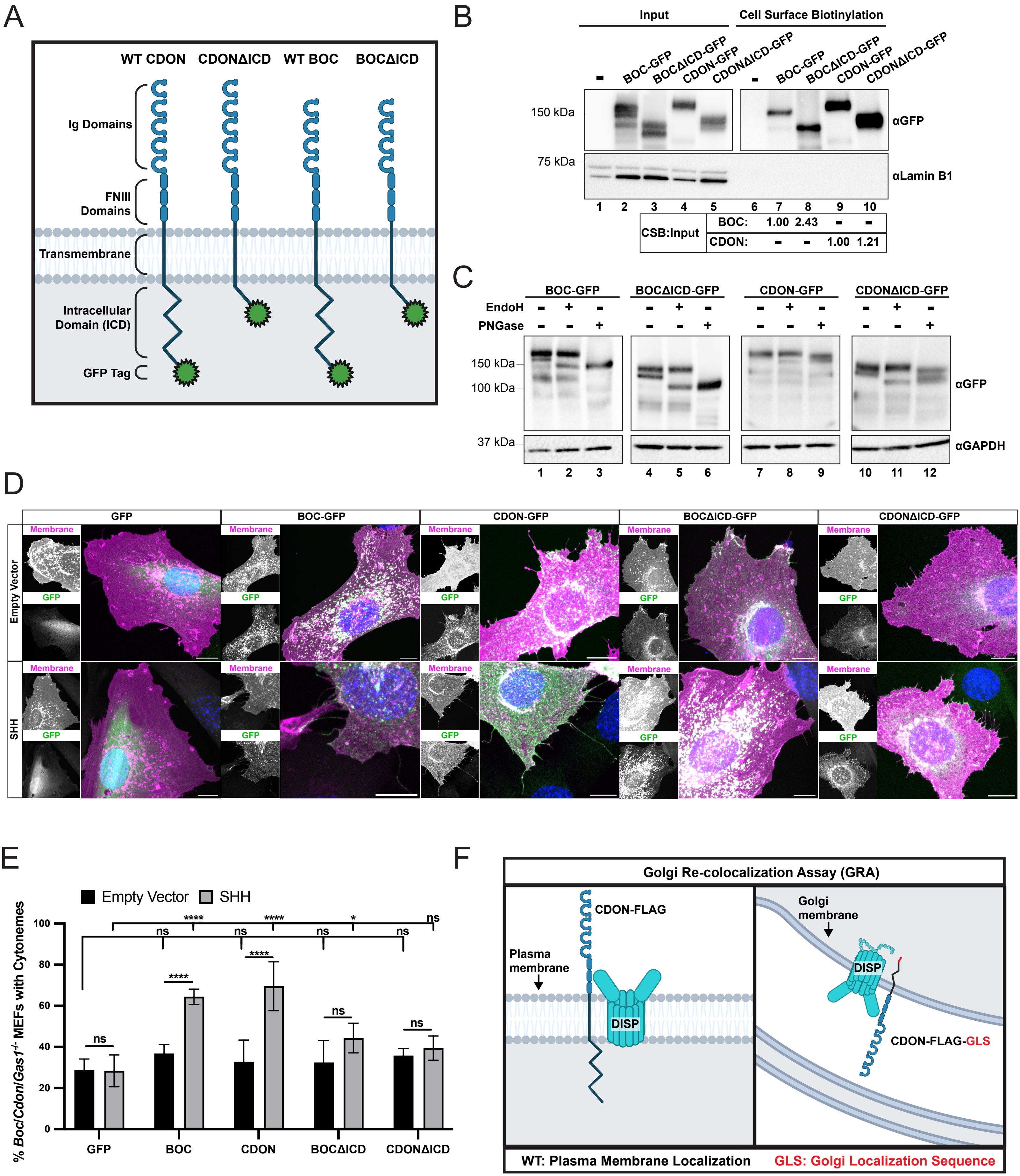

A
WT CDON
CDONΔICD
WT BOC
BOCΔICD
Ig Domains
FNIII Domains
Transmembrane
Intracellular Domain (ICD)
GFP Tag
B
Input
Cell Surface Biotinylation
BOC-GFP
BOCΔICD-GFP
CDON-GFP
CDONΔICD-GFP
150 kDa
75 kDa
αGFP
αLamin B1
1 2 3 4 5 6 7 8 9 10
CSB:Input
BOC: 1.00 2.43
CDON: 1.00 1.21
C
BOC-GFP
BOCΔICD-GFP
CDON-GFP
CDONΔICD-GFP
EndoH
PNGase
150 kDa
100 kDa
37 kDa
αGFP
αGAPDH
1 2 3 4 5 6 7 8 9 10 11 12
D
GFP
BOC-GFP
CDON-GFP
BOCΔICD-GFP
CDONΔICD-GFP
Empty Vector
SHH
Membrane
GFP
E
Empty Vector
SHH
% Boc/Cdon/Gas1-/- MEFs with Cytonemes
0 20 40 60 80 100
GFP
BOC
CDON
BOCΔICD
CDONΔICD
ns
****
*
F
Golgi Re-colocalization Assay (GRA)
CDON-FLAG
Plasma membrane
DISP
Golgi membrane
CDON-FLAG-GLS
WT: Plasma Membrane Localization
GLS: Golgi Localization Sequence

Fig. S5.
(**A**) BOC and CDON are transmembrane proteins with tandem N-terminal Immunoglobulin-like (Ig) domains, Fibronectin type III (FNIII) domains, and intracellular domains (ICD). C-terminally GFP-tagged constructs lacking the intracellular domains were generated (ΔICD). Created in BioRender. Daly, C. (2026) https://BioRender.com/8r0dpb8. (**B**) Cell surface biotinylation (CSB) of NIH3T3 cells expressing GFP-tagged BOC, BOCΔICD, CDON, or CDONΔICD. Biotinylated proteins were isolated from lysates using Streptavidin-conjugated beads and detected via immunoblotting. Lamin B1 serves as an intracellular loading control. Relative CSB:Input levels were calculated based on the blot shown. (**C**) HEK293T cell lysates expressing GFP-tagged BOC, BOCΔICD, CDON, or CDONΔICD were treated with EndoH or PNGase glycosidases. N-linked glycosylation was detected through molecular weight shifts following glycan removal. GAPDH serves as a loading control. (**D**) *Boc*/*Cdon*/*Gas1*$^{-/-}$ MEFs stably expressing either MSCV-Hygro (Empty Vector) or MSCV-Hygro-SHH-FL (SHH) were transfected with membrane-mCherry (magenta) and either GFP, WT BOC- or CDON-GFP, or BOCΔICD- or CDONΔICD-GFP (green). DAPI is shown in blue. Scale bars represent 10 µm. (**E**) Cytoneme occurrence rates were quantified on n = 139-351 cells per condition acquired over three biological replicates. (**F**) Golgi re-colocalization assay (GRA): Flag-tagged hCDON localizes at the plasma membrane with V5-DISP-HA (left panel). CDON was generated with a C-terminal Golgi localization sequence (CDON-FLAG-GLS). CDON-FLAG-GLS is retained in the Golgi apparatus and interactors such as V5-DISP-HA are re-localized to the Golgi apparatus (right panel). Created in BioRender. Daly, C. (2026) https://BioRender.com/qi95s2h. For all panels, immunoblots are representative of at least 2 biological replicates. Data shown as mean ± SD. Significance was calculated using One-Way ANOVA. *$p < 0.05$, ****$p < 0.0001$, ns = not significant.

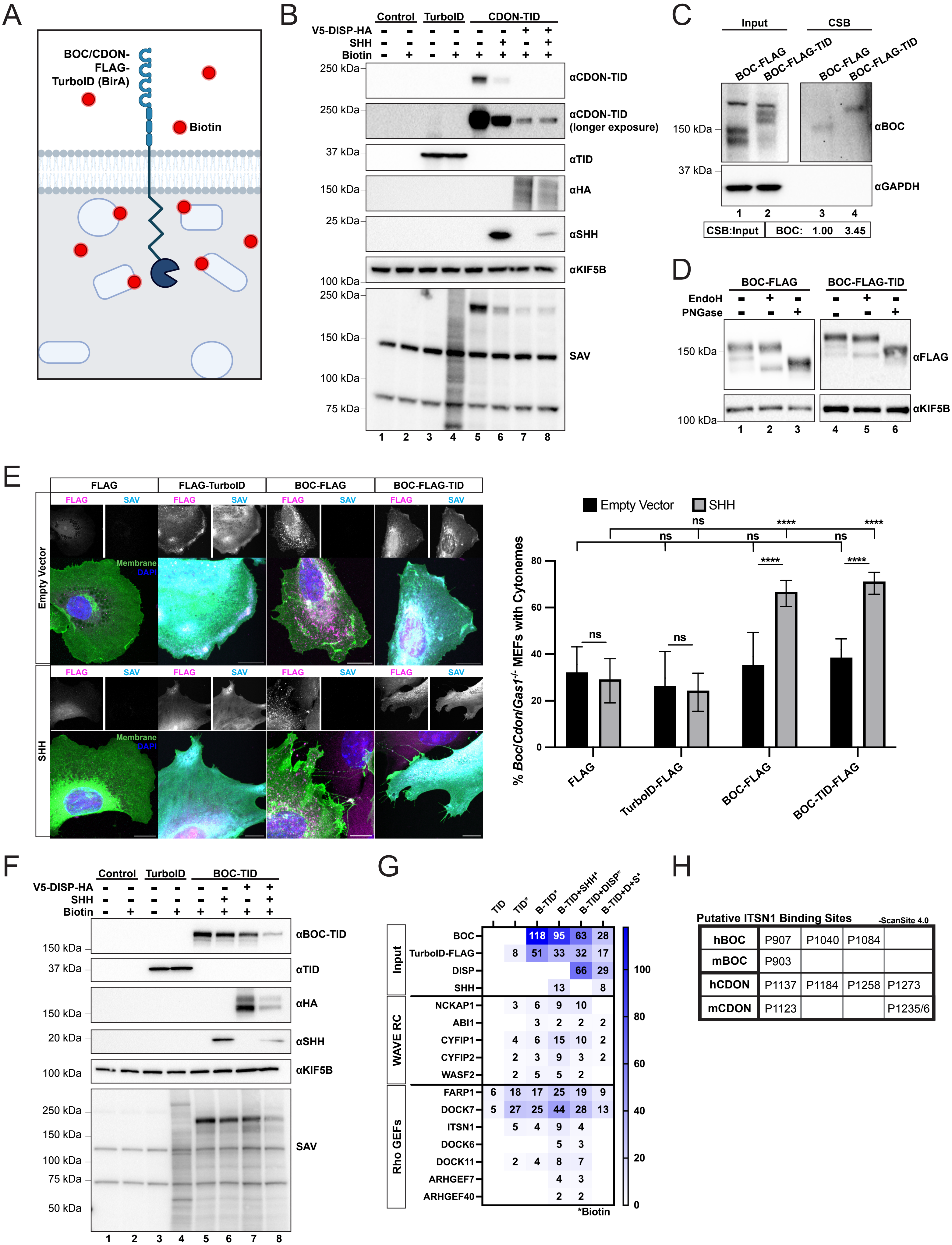

A
BOC/CDON-FLAG-TurboID (BirA)
Biotin
B
Control
TurboID
CDON-TID
V5-DISP-HA
SHH
Biotin
250 kDa
αCDON-TID
αCDON-TID (longer exposure)
37 kDa
αTID
150 kDa
αHA
25 kDa
αSHH
αKIF5B
100 kDa
75 kDa
SAV
1 2 3 4 5 6 7 8
C
Input
CSB
BOC-FLAG
BOC-FLAG-TID
150 kDa
αBOC
37 kDa
αGAPDH
1 2 3 4
CSB:Input
BOC: 1.00 3.45
D
BOC-FLAG
BOC-FLAG-TID
EndoH
PNGase
150 kDa
αFLAG
αKIF5B
100 kDa
1 2 3 4 5 6
E
FLAG
FLAG-TurboID
BOC-FLAG
BOC-FLAG-TID
SAV
Membrane
DAPI
Empty Vector
SHH
Empty Vector
SHH
ns
****
% Boc/Cdon/Gas1-/- MEFs with Cytonemes
0 20 40 60 80
FLAG
TurboID-FLAG
BOC-FLAG
BOC-TID-FLAG
F
Control
TurboID
BOC-TID
V5-DISP-HA
SHH
Biotin
150 kDa
αBOC-TID
37 kDa
αTID
αHA
20 kDa
αSHH
100 kDa
αKIF5B
250 kDa
50 kDa
SAV
1 2 3 4 5 6 7 8
G
TID
TID*
B-TID*
B-TID+SHH*
B-TID+DISP*
B-TID+D+S*
Input
BOC 118 95 63 28
TurboID-FLAG 8 51 33 32 17
DISP 66 29
SHH 13 8
WAVE RC
NCKAP1 3 6 9 10
ABI1 3 2 2 2
CYFIP1 4 6 15 10 2
CYFIP2 2 3 9 3 2
WASF2 2 5 5 2
Rho GEFs
FARP1 6 18 17 25 19 9
DOCK7 5 27 25 44 28 13
ITSN1 5 4 9 4
DOCK6 5 3
DOCK11 2 4 8 7
ARHGEF7 4 3
ARHGEF40 2 2
*Biotin
0 20 40 60 80 100
H
Putative ITSN1 Binding Sites
-ScanSite 4.0
hBOC P907 P1040 P1084
mBOC P903
hCDON P1137 P1184 P1258 P1273
mCDON P1123 P1235/6

Fig. S6.

(**A**) BOC and CDON constructs were generated with a C-terminal BirA/TurboID biotin ligase. Biotin treatment of cells allows the TurboID enzyme to biotinylate nearby molecules for isolation and identification. Created in BioRender. Daly, C. (2026) https://BioRender.com/88myv0o. (**B**) CDON-FLAG-TurboID shows poor expression and has weak biotinylation activity. (**C**) Cell surface biotinylation (CSB) of NIH3T3 cells expressing FLAG- or FLAG-TurboID-tagged BOC. Surface proteins were isolated using Streptavidin-conjugated beads. GAPDH is the loading control. Relative CSB:Input levels were calculated based on the blot shown. (**D**) HEK293T cell lysates expressing FLAG- or FLAG-TurboID-tagged BOC were treated with EndoH or PNGase. Molecular weight shifts following treatment with glycosidases are indicative of N-linked glycosylation. KIF5B is the loading control. (**E**) *Boc*/*Cdon*/*Gas1*$^{-/-}$ MEFs stably expressing either MSCV-Hygro (Empty Vector) or MSCV-Hygro-SHH-FL were transfected with membrane-pHluorin (green), and FLAG, FLAG-TurboID, BOC-FLAG, or BOC-FLAG-TurboID (magenta). Cells were treated with biotin and fixed. Biotinylation was detected using Streptavidin (SAV; cyan), DAPI is blue. Scale bars = 10 µm. Cytoneme occurrence rates were quantified over three biological replicates (n = 121-361 cells/condition). Data shown as mean ± SD. Significance was calculated using One-Way ANOVA. ****$p < 0.0001$, ns = not significant. (**F**) *Boc*/*Cdon*/*Disp1/Ptch1/Ptch2/Smo*$^{-/-}$ MEFs were transfected with either FLAG-TurboID or BOC-FLAG-TurboID and V5-DISP-HA and/or SHH. Cells were treated with Biotin or DMSO vehicle. Lysates were immunoblotted with anti-BirA/TurboID, anti-HA, anti-SHH, and Streptavidin to ensure biotinylation. KIF5B is the loading control. Biotinylated proteins were isolated using Streptavidin-conjugated beads and identified using liquid chromatography tandem mass spectrometry (LC-MS/MS) spectral counting-based quantification. (**G**) Spectral counts for validated interactor WAVE Regulatory Complex (RC) members and candidate interactor Rho Guanine nucleotide Exchange Factors (GEFs) are listed, including the spectral counts for TurboID (TID) alone or TurboID treated with biotin (TID*). Blank spaces indicate zero spectral counts. (**H**) Putative ITSN1 binding sites for human (h) or mouse (m) BOC/CDON (ScanSite 4.0).

A

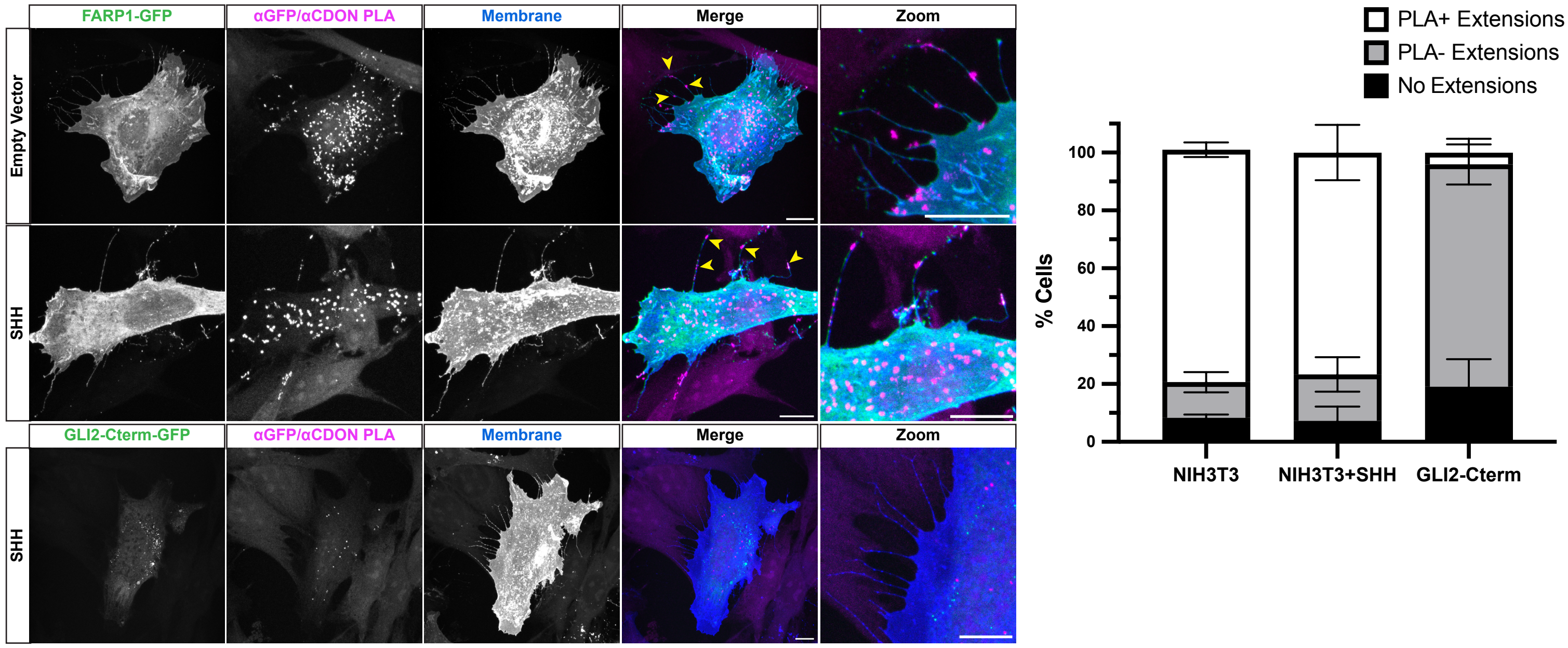
FARP1-GFP
αGFP/αCDON PLA
Membrane
Merge
Zoom
Empty Vector
SHH
GLI2-Cterm-GFP
αGFP/αCDON PLA
Membrane
Merge
Zoom
SHH
PLA+ Extensions
PLA- Extensions
No Extensions
% Cells
100
80
60
40
20
0
NIH3T3
NIH3T3+SHH
GLI2-Cterm

B

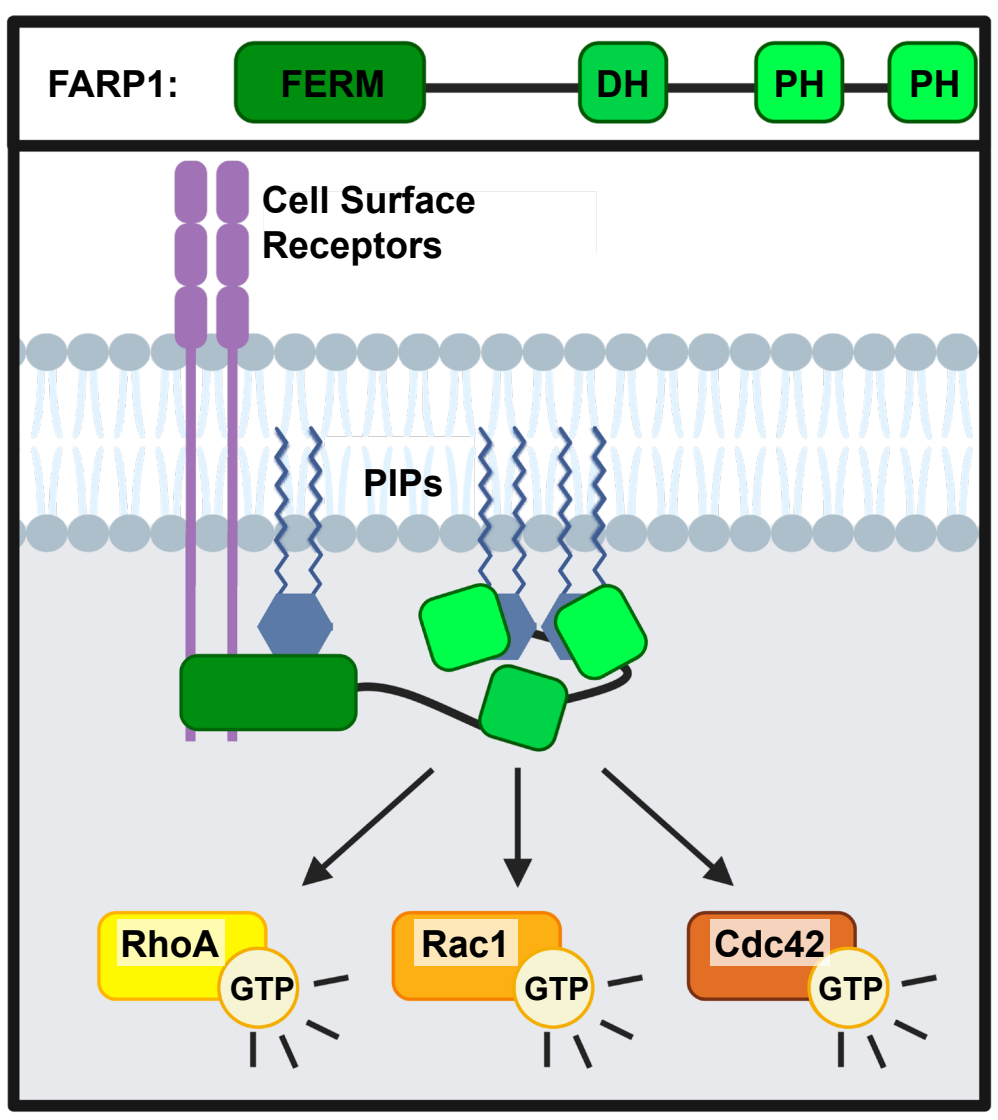
FARP1:
FERM
DH
PH
PH
Cell Surface
Receptors
PIPs
RhoA
GTP
Rac1
GTP
Cdc42
GTP

Fig S7.
(**A**) NIH3T3 cells stably expressing MSCV-Hygro (Empty Vector) or MSCV-Hygro-SHH-FL were transiently transfected with CDON-FLAG and either FARP1-GFP or GLI2-C term-GFP (green). Proximity ligation assays (PLA; magenta) were performed using primary antibodies against CDON and GFP. Membrane-BFP (Blue) shows cellular extensions that contain PLA between CDON and FARP1 but are absent for PLA between CDON and GLI2-C term. Scale bars = 10 µm. Percentage of cells with PLA-positive (white) or PLA-negative (gray) extensions and cells without extensions (black) were quantified over 3 biological replicates totaling 151-238 cells per condition. (**B**) FARP1 (green) is a Dbl-family Guanine nucleotide exchange factor (GEF) with an N-terminal 4.1, ezrin, radixin, and moesin (FERM) domain that can interact with proteins such as some cell surface receptors (purple). FARP1 has tandem C-terminal Pleckstrin Homology (PH) domains that can interact with phosphatidylinositol phosphates (PIPs; blue) at the plasma membrane. The Dbl Homology (DH) domain is responsible for activating Rho GTPases (yellow/orange), and has the capability to activate RhoA, Rac1, and/or Cdc42, depending on cellular context. Created in BioRender. Daly, C. (2026) https://BioRender.com/gkq5s0y

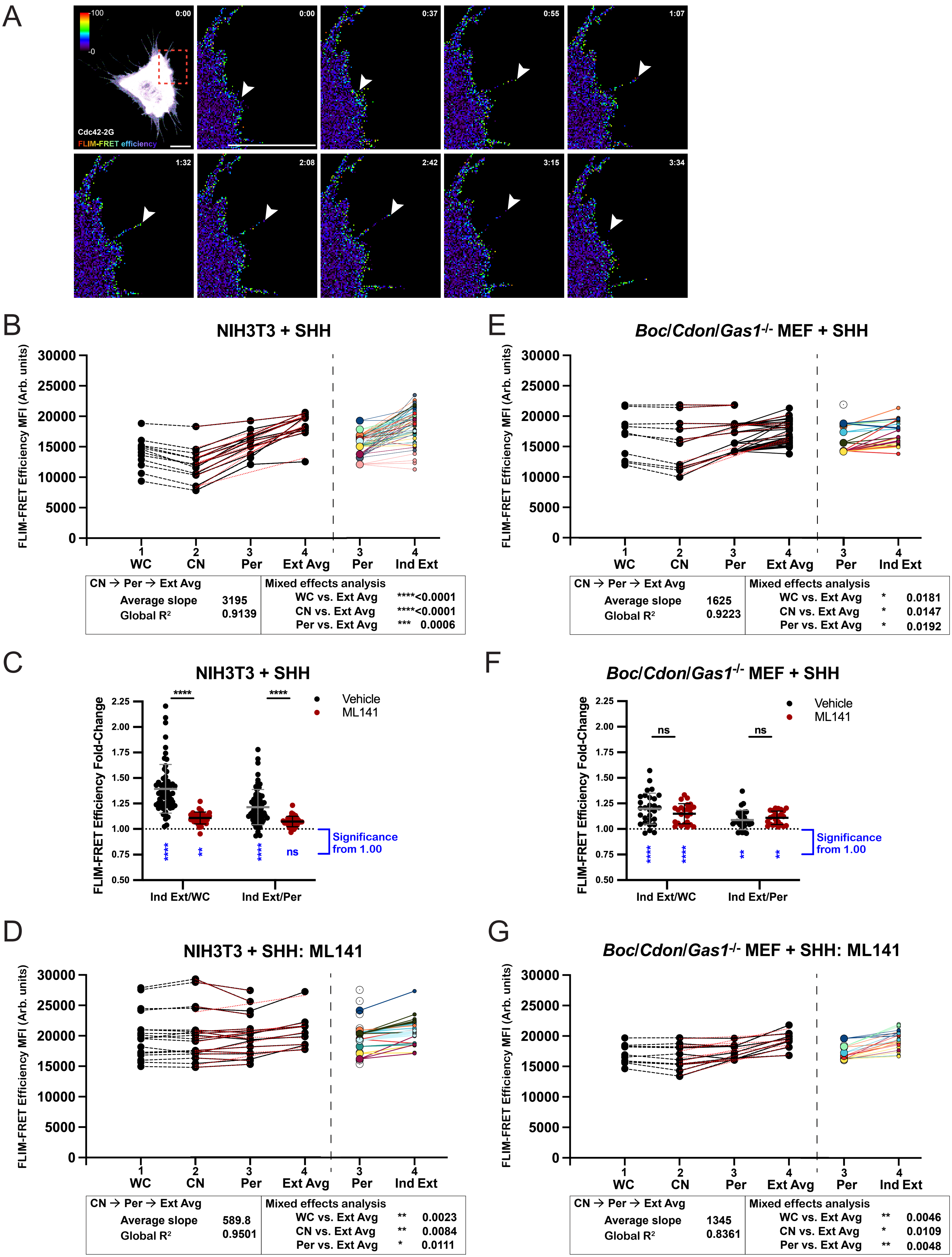
A
100
0
0:00
0:37
0:55
1:07
1:32
2:08
2:42
3:15
3:34
Cdc42-2G
FLIM-FRET efficiency
B
NIH3T3 + SHH
FLIM-FRET Efficiency MFI (Arb. units)
30000
25000
20000
15000
10000
5000
0
1 WC
2 CN
3 Per
4 Ext Avg
3 Per
4 Ind Ext
CN → Per → Ext Avg
Average slope 3195
Global R² 0.9139
Mixed effects analysis
WC vs. Ext Avg ****<0.0001
CN vs. Ext Avg ****<0.0001
Per vs. Ext Avg *** 0.0006
E
Boc/Cdon/Gas1-/- MEF + SHH
CN → Per → Ext Avg
Average slope 1625
Global R² 0.9223
Mixed effects analysis
WC vs. Ext Avg * 0.0181
CN vs. Ext Avg * 0.0147
Per vs. Ext Avg * 0.0192
C
NIH3T3 + SHH
FLIM-FRET Efficiency Fold-Change
2.25
2.00
1.75
1.50
1.25
1.00
0.75
0.50
Vehicle
ML141
****
****
**
****
ns
Significance from 1.00
Ind Ext/WC
Ind Ext/Per
F
Boc/Cdon/Gas1-/- MEF + SHH
ns
ns
****
****
**
**
Significance from 1.00
D
NIH3T3 + SHH: ML141
CN → Per → Ext Avg
Average slope 589.8
Global R² 0.9501
Mixed effects analysis
WC vs. Ext Avg ** 0.0023
CN vs. Ext Avg ** 0.0084
Per vs. Ext Avg * 0.0111
G
Boc/Cdon/Gas1-/- MEF + SHH: ML141
CN → Per → Ext Avg
Average slope 1345
Global R² 0.8361
Mixed effects analysis
WC vs. Ext Avg ** 0.0046
CN vs. Ext Avg * 0.0109
Per vs. Ext Avg ** 0.0048

Fig S8.
(**A**) Time series of NIH3T3 cell stably expressing MSCV-Hygro-SHH-FL (NIH3T3 + SHH) transfected with Cdc42-2G biosensor. Rainbow pseudocoloring corresponds to FLIM-FRET efficiency (0-100%) and Cdc42 activity levels. Cdc42 activity is high at extension tips during outgrowth and decreases prior to retraction. Scale bars are 10 µm and time stamp indicates min:sec. (**B**-**G**) FLIM-FRET mean fluorescence intensity (MFI) was measured in the indicated ROIs: whole cell (WC), ~5 µm of cell periphery (Per), central node (CN), and cellular extensions (Ext) of NIH3T3 + SHH cells (**B**-**D**) or *Boc*/*Cdon*/*Gas1*$^{-/-}$ + SHH MEFs (**E**-**G**) treated with vehicle (**B**,**E**) or Cdc42 inhibitor ML141 (**D**,**G**). Both the average MFI for all extensions per cell (Ext Avg) and the MFI value for each individual extension (Ind Ext) are shown, with Ind Ext color-coded based on the cell of origin. Least squares analysis was conducted on the MFI values from CN to Per to Ext Avg (black solid lines) to generate regressions (red dotted lines) for each cell. Corresponding WC MFI values are connected to CN with black dotted lines. The average slope and global R^2 are reported, with higher slopes corresponding to higher Cdc42 activity in the Ext compared to the CN and Per. Mixed effects analyses were performed to determine statistical significance between Ext Avg and the other ROIs. FLIM-FRET efficiencies derived from NIH3T3 + SHH cells (**C**) and *Boc*/*Cdon*/*Gas1*$^{-/-}$ + SHH MEFs (**F**) were normalized as fold-change between ROIs. Ind Ext fold-change from WC and Per is presented. Significance between vehicle and ML141-treated cells is represented using Welch's t-test (black asterisks), and significance from a baseline fold-change of 1.0 is represented using one-way ANOVA (blue asterisks). *$p < 0.05$, **$p < 0.01$, ***$p < 0.001$, ****$p < 0.0001$, ns = not significant.

Movie S1.
Live imaging of a E9.5/26 somite *Shh-Cre*;*mTmG* embryo section reveals many long cellular extensions emanating from the SHH-producing floor plate. Membrane-tomato is in blue, membrane-GFP is in yellow. Each panel represents varying Z-depths into the tissue, corresponding to Fig. 2B. Arrowhead colors correspond to Z slice of origin, and white-outlined arrowheads indicate extensions that are observed in multiple Z plane panels. Time stamp indicates min:sec, scale bar = 10 µm.

Movie S2.
Live imaging of neural tube sections from two additional E9.5/25 somite stage *Shh-Cre*;*mTmG* embryos serve as supplementary examples of extensions formed by SHH-producing floor plate cells; corresponding to Fig. 2D-E. Arrowheads mark extensions observed during each movie. Membrane-tomato is in blue, membrane-GFP is in yellow; time stamp indicates min:sec, scale bar = 10 µm.

Movie S3.
Live imaging of E9.5/21-27 somite stage mutant embryos reveals a decrease in extension numbers and lengths in *Shh-Cre*;*Cdon*$^{flox/+}$;*mTmG* embryos (top panels) and a further loss of extensions in *Shh-Cre*;*Cdon*$^{flox/flox}$;*mTmG* embryos (bottom panels), regardless of *Boc* expression. Movies correspond to Fig. 2C; arrowhead colors correspond to genotype. Membrane-tomato is in blue, membrane-GFP is in yellow; time stamp indicates min:sec, scale bar = 10 µm.

Movie S4.
Live imaging of E9.5/somite stage 24-26 mutant embryos show floor plate extensions in *Shh-Cre*;*Cdon*$^{flox/+}$;*mTmG* embryos (top panels: three Z-depths; bottom left panel) and fewer extensions in *Shh-Cre*;*Cdon*$^{flox/flox}$;*mTmG* embryos (bottom: middle and right panels). Movies correspond to Fig. 2D-E; arrowhead colors correspond to genotype. Membrane-tomato is in blue, membrane-GFP is in yellow; time stamp indicates min:sec, scale bar = 10 µm.

Movie S5.
Live imaging of NIH3T3 cell stably expressing MSCV-Hygro-SHH-FL transfected with Cdc42-2G biosensor. Rainbow pseudocoloring corresponds to FLIM-FRET efficiency (0-100%) and Cdc42 activity levels. ML141 treatment (right panel) abrogates Cdc42 activity dynamic range. Movie corresponds to Fig. 7A; time stamp indicates hh:min:sec.

Movie S6.
Live imaging of *Boc*/*Cdon*/*Gas1*$^{-/-}$ MEF stably expressing MSCV-Hygro-SHH-FL transfected with Cdc42-2G biosensor. Cdc42 activity (FLIM-FRET efficiency, 0-100%) is in rainbow. ML141 treatment (right panel) attenuates Cdc42 activity and brings efficiency to baseline levels. Movie corresponds to Fig. 7B; time stamp indicates hh:min:sec.

Data S1. (separate file)
BOC-TurboID proximity labeling.
